# Design and synthesis of nucleolipids as possible activated precursors for oligomer formation via intramolecular catalysis: stability study and supramolecular organization

**DOI:** 10.1186/s13322-014-0005-3

**Published:** 2014-12-09

**Authors:** Kishore Lingam Gangadhara, Puneet Srivastava, Jef Rozenski, Henri-Philippe Mattelaer, Volker Leen, Wim Dehaen, Johan Hofkens, Eveline Lescrinier, Piet Herdewijn

**Affiliations:** Medicinal Chemistry, Rega Institute for Medical Research, KU Leuven, Leuven, Minderbroederstraat-10, 3000 Leuven, Belgium; Department of Chemistry, Molecular Design and Synthesis, KU Leuven, Leuven, Belgium; Department of Chemistry, Molecular Imaging and Photonics, KU Leuven, Leuven, Belgium

**Keywords:** Nucleolipid, Vesicles, Hydroxy fatty acids, Protocell, Chemical stability, Supramolecular assembly, Intramolecular catalysis, Fluorescence microscopy, BODIPY, NMR stability study

## Abstract

**Background:**

Fatty acid vesicles are an important part of protocell models currently studied. As protocells can be considered as pre-biological precursors of cells, the models try to contribute to a better understanding of the (cellular) origin of life and emphasize on 2 major aspects: compartmentalization and replication. It has been demonstrated that lipid-based membranes are amenable to growth and division (shell replication). Furthermore compartmentalization creates a unique micro-environment in which biomolecules can accumulate and reactions can occur. Pioneering research by Sugawara, Deamer, Luisi, Szostak and Rasmussen gave more insight in obtaining autocatalytic, self-replicating vesicles capable of containing and reproducing nucleic acid sequences (core replication). Linking both core and shell replication is a challenging feat requiring thorough understanding of membrane dynamics and (auto)catalytic systems. A possible solution may lie in a class of compounds called nucleolipids, who combine a nucleoside, nucleotide or nucleobase with a lipophilic moiety. Early contributions by the group of Yanagawa mentions the prebiotic significance (as a primitive helical template) arising from the supramolecular organization of these compounds. Further contributions, exploring the supramolecular scope regarding phospoliponucleosides (e.g. 5’-dioleylphosphatidyl derivatives of adenosine, uridine and cytidine) can be accounted to Baglioni, Luisi and Berti. This emerging field of amphiphiles is being investigated for surface behavior, supramolecular assembly and even drug ability.

**Results:**

A series of α/β-hydroxy fatty acids and α-amino fatty acids, covalently bound to nucleoside-5′-monophosphates via a hydroxyl or amino group on the fatty acid was examined for spontaneous self-assembly in spherical aggregates and their stability towards intramolecular cleavage. Staining the resulting hydrophobic aggregates with BODIPY-dyes followed by fluorescent microscopy gave several distinct images of vesicles varying from small, isolated spheres to higher order aggregates and large, multimicrometer sized particles. Other observations include rod-like vesicle precursors. NMR was used to assess the stability of a representative sample of nucleolipids. 1D ^31^P NMR revealed that β-hydroxy fatty acids containing nucleotides were pH-stable while the α-analogs are acid labile. Degradation products identified by [^1^H-^31^P] heteroTOCSY revealed that phosphoesters are cleaved between sugar and phosphate, while phosphoramidates are also cleaved at the lipid-phosphate bond. For the latter compounds, the ratio between both degradation pathways is influenced by the nucleobase moiety. However no oligomerization of nucleotides was observed; nor the formation of 3′-5′-cyclic nucleotides, possible intermediates for oligonucleotide synthesis.

**Conclusions:**

The nucleolipids with a deoxyribose sugar moiety form small or large vesicles, rod-like structures, vesicle aggregates or large vesicles. Some of these aggregates can be considered as intermediate forms in vesicle formation or division. However, we could not observe nucleotide polymerization or cyclic nucleotide function of these nucleolipids, regardless of the sugar moiety that is investigated (deoxyribose, ribose, xylose). To unravel this observation, the chemical stability of the constructs was studied. While the nucleolipids containing β-hydroxy fatty acids are stable as well in base as in acid circumstances, others degraded in acidic conditions. Phosphoramidate nucleolipids hydrolyzed by P-N as well as P-O bond cleavage where the ratio between both pathways depends on the nucleobase. Diester constructs with an α-hydroxy stearic acid degraded exclusively by hydrolysis of the 5′-O-nucleoside ester bond. As the compounds are too stable and harsh conditions would destruct the material itself, more reactive species such as lipid imidazolates of nucleotides need to be synthesized to further analyze the potential polymerization process.

**Graphical Abstract:**
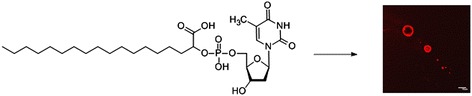
Vesicle information of a nucleolipid consisting of a nucleoside 5'-monophosphate and a α-hydroxy fatty acid.

## Background

Fatty acid vesicles are an important part of protocell models currently studied [1,2]. As protocells can be considered as pre-biological precursors of cells [3], the models try to contribute to a better understanding of the (cellular) origin of life and emphasize on 2 major aspects: compartmentalization and replication [2,4-6]. It has been demonstrated that lipid-based membranes are amenable to growth and division [1,7]. Small unilamellar vesicles divide after micelle addition [8]. Autocatalytic self-replicating micelles are formed from amphiphiles generated from the alkaline hydrolysis of ethyl caprylate (shell replication) [4]. Furthermore compartmentalization creates a unique micro-environment in which biomolecules can accumulate [9,10] and reactions can occur [11]. Pioneering research by Sugawara [12], Deamer [13], Luisi [4], Szostak [7,14,15] and Rasmussen [16] gave more insight in obtaining autocatalytic, self-replicating vesicles capable of containing and reproducing nucleic acid sequences (core replication).

Linking both core and shell replication is a challenging feat requiring thorough understanding of membrane dynamics [7] and (auto)catalytic systems [4,17]. A possible solution may lie in a class of compounds called nucleolipids, who combine a nucleoside, nucleotide or nucleobase with a lipophilic moiety. Early contributions by the group of Yanagawa [18] mentions the prebiotic significance (as a primitive helical template) arising from the supramolecular organization of these compounds. Further contributions, exploring the supramolecular scope regarding phospoliponucleosides (e.g. 5’-dioleylphosphatidyl derivatives of adenosine, uridine and cytidine) can be accounted to Baglioni, Luisi and Berti. This emerging field of amphiphiles is being investigated for surface behavior, supramolecular assembly and even drug ability [19,20]. Besides improving permeability, modifying medicinally active nucleosides or nucleotides with long alkyl chains has proven (also by our group) to be an adequate prodrug tactic [10,21].

**Scheme 1:**
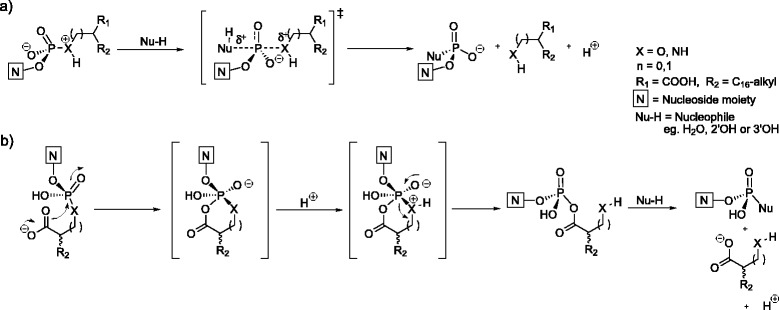
**Rationale behind the design of a new series of nucleolipid containing hydroxy/amino fatty acid with a free α/β carboxyl group.** Plain acid promoted nucleophilic cleavage **(a)** and by carboxyl group mediated, intramolecular catalyzed nucleophilic cleavage **(b)**.

Now we designed of a series of nucleolipids as possible activated precursors for obtaining oligonucleotides. Besides its role as supramolecular recognition element ensuring the vicinity of the nucleophilic 2′- and 3′-hydroxyl groups and the electrophilic (activated) phosphate, it is necessary that the lipid part of the conjugate is also a good leaving group. This may be achieved by intramolecular catalysis; as we have recently demonstrated that a carboxylic acid function introduced in α-position of a phosphoramidate or phosphodiester group may help in catalyzing the cleavage of the phosphoramidate or phosphodiester bonds. This occurs by means of a cyclic intermediate that forms under (mild) acidic conditions (Scheme 1b). One must also consider the competing acidic hydrolysis (Scheme 1a) and cleavage of the ester bond between nucleoside and phosphate (not depicted). Previous results have shown that the cleavage ratio of Nucleoside-O-P and P-X-leaving group depends on the nature of the latter bond, the leaving group (with or without nearby carboxyl group), nucleobase and pH (a more detailed discussion of these factors can be found in the work of Maiti et al [22]).

Depending on the cleaved bond, this might lead to oligonucleotide formation due to the leaving group properties of the lipid moiety or through the formation of cyclic nucleotides, (e.g. 3′-5′ cyclic GMP) which are able to polymerize in water to give short RNA fragments [23]. As the properties of the phospholipids with and without nucleoside are different, the potential of obtaining a dynamic system is present.

**Figure 1:**
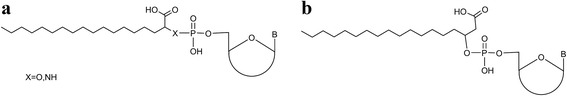
**General structure of nucleolipids studied in this manuscript: a: α-hydroxystearic acid (X = O), α-aminostearic acid (X = NH) and b: β-hydroxystearic acid.**

Here, we have investigated the potential of α/β-hydroxy fatty acids and α-amino fatty acids, covalently bound to nucleoside-5′-monophosphates via a hydroxyl or an amino group on the fatty acid (Figure 1) to spontaneous self-assemble in spherical aggregates. Their stability towards intramolecular cleavage was examined and thus their ability to function as (activated) monomer for oligonucleotide synthesis was assessed.

## Results and discussion

### Chemistry

Several types of phospholipid conjugates of nucleotides were synthesized as represented by structure A and B (Figure 1). A stearic acid scaffold should provide an optimal balance between membrane fluidity and sufficient permeability [2]. A nucleoside moiety consists of either a (deoxy)ribofuranose or a xylofuranose linked to thymine or adenine; thus creating amphiphiles with large, polar head groups.

**Scheme 2:**
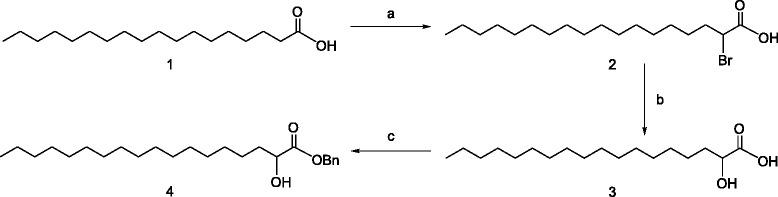
**Synthesis of (±)-α hydroxy stearate. a)** PBr_3_, Br_2_, 95°C, 6 h; **b)** 2 M NaOH, 85°C, 2 h; **c)** Et_3_N, BnBr, TBAI, toluene, reflux 12 h.

**Scheme 3:**
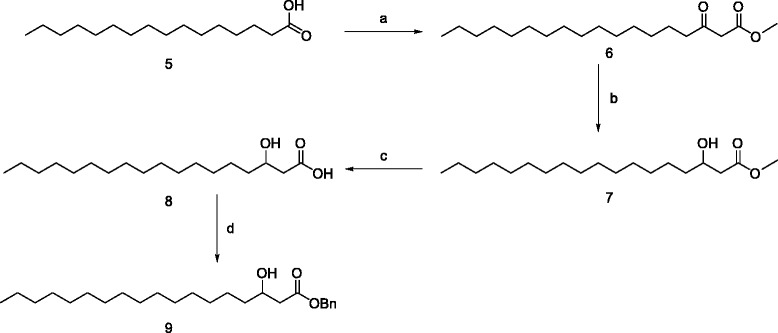
**Synthesis of (±)-β hydroxy stearate. a)** 1,1′-carbonyldiimidazole, magnesium methylmalonate, THF, rt 24 h; **b)** NaBH_4_, EtOH, rt,15 min; **c)** 1 N NaOH, 15 min rt; **d)** Et_3_N, BnBr, TBAI, toluene, reflux 12 h.

The (±)-α-hydroxy stearic acid **3** is prepared in two steps from stearic acid **1** by bromination [24] using Hell-Volhard-Zelinsky condition giving compound **2** followed by hydrolysis in 88% overall yield (Scheme 2). The (±)-β-Hydroxy stearic acid **8** is synthesized by the procedure described by Masamune [25,26], which involves homologation of palmitic acid **5** [27] using *in situ* generated magnesium monomethylmalonate to a preformed acyl imidazole to produce the β-keto stearic acid methyl ester, which is reduced [28] with sodium borohydride in ethanol providing **7**. Saponification with 1 N NaOH resulted in the formation of (±)-β-hydroxy stearic acid **8** in 77% overall yield (Scheme 3).

The benzyl esters of α-hydroxy stearate **4** was synthesized [29] by heating α-hydroxystearic acid **3**, with benzyl bromide in presence of triethylamine, and catalytic amount of TBAI in toluene for 12 h in a yield of 56%. The benzyl ester of β-hydroxy stearate **9** is prepared in a similar way.

**Scheme 4:**
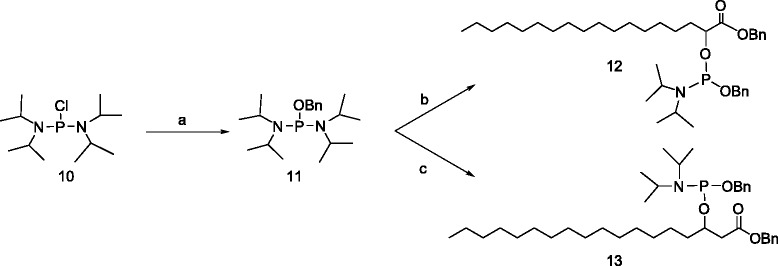
**Synthesis of the phosphoramidite reagents. a)** BnOH, Et_3_N, Et_2_O; **b)** 4, dry DCM, 1H-Tetrazole at 0°C, 15 min at rt; **c)** 9, dry DCM, 1H-Tetrazole at 0°C, 15 min at rt.

The phosphoramidite approach is used for the synthesis of the lipid-nucleotide conjugates. The phosphoramidites of (±)-α- **12** and (±)-β-hydroxy benzyl stearate **13** (Scheme 4) were prepared by reaction of **4** and **9**, respectively, with the phosphoramidite reagent **11** (Scheme 4), which was obtained by reaction of bis(diisopropylamino)chlorophosphine [30] **10** with benzyl alcohol.

**Scheme 5:**
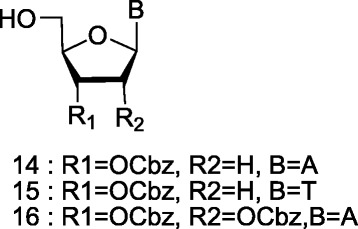
**Sugar-protected adenine and thymine nucleoside.**

The protected 2′-deoxyadenosine **14** and adenosine **16** were prepared in a similar manner as previously described [31,32]. Likewise, protected thymidine **15** was prepared according to a previously reported procedure [31,32] (Scheme 5).

**Scheme 6:**
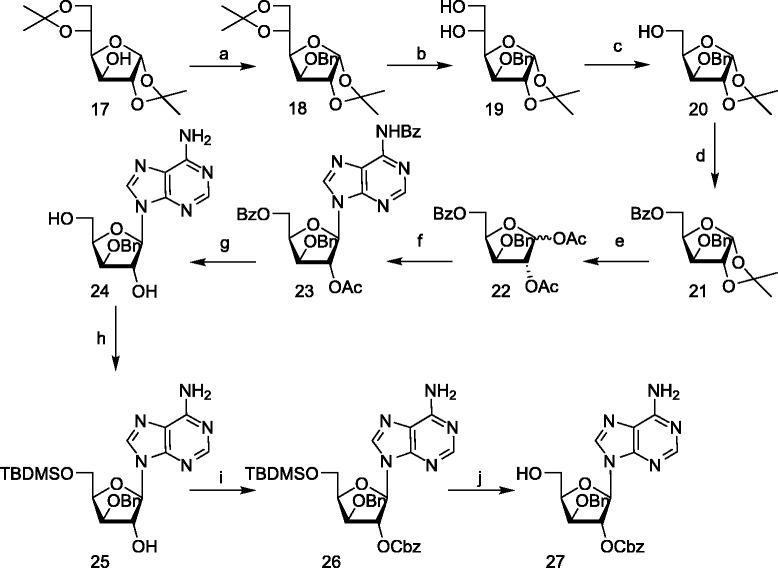
**Synthesis of the protected xylofuranosyl nucleoside. a)** NaH, DMF, BnBr, 0°C - rt 24 h; **b)** 1:1 methanol/1% aqueous sulfuric acid; **c)** NaIO_4_, H_2_O rt, NaBH_4_, EtOH 0°C - rt 2 h; **d)** benzoyl chloride, pyr; **e)** acetic acid, acetic anhydride, H_2_SO_4_; **f)** SnCl_4_, acetonitrile, rt 5 h; **g)** NH_4_OH, MeOH, rt, 2d; **h)** dry DMF, Imidazole, TBDMSCl. 24 h, rt; **i)** dry DCM, DMAP, CbzCl, 72 h, rt; **j)** HF in pyridine, pyridine, rt 4 h.

The 2′, 3′-O-protected xylofuranose derivative **27** with an adenine base moiety (Scheme 6) was prepared, starting from commercially available diacetone-D-glucose **17**. Benzoylation of **17** was carried out using sodium hydride and benzyl bromide in dry dimethylformamide. Selective hydrolysis of the terminal isopropylidene group followed by oxidation with sodium periodate, and borohydride reduction, afforded the 1,2-acetonide **20**, in 88% yield over three steps. After benzoylation of **20** [33,34] and treatment of **21** with Ac_2_O/H_2_SO_4_, a mixture of the anomers of 1,2-di-*O*-acetyl-3-*O*-benzyl-5-*O*-benzoyl-D-xylofuranose **22** was obtained. Condensation of **22** with N^6^-benzoyladenine [35,36], provided the desired β-anomer **23** in 86% yield.

Deprotection of **23** was carried out by using saturated methanolic ammonia to give the nucleoside **24** in 68% yield. The selective silylation of the 5′-hydroxyl group of compound **24** was carried out with TBDMSCl, imidazole in dry DMF to obtain nucleoside **25**. The 2′-hydroxyl group of compound **25** was protected with a carbobenzyloxy group. Finally, the 5′-O-TBDMS group is removed with 1 M TBAF in dry THF to obtain compound **27**.

**Scheme 7:**
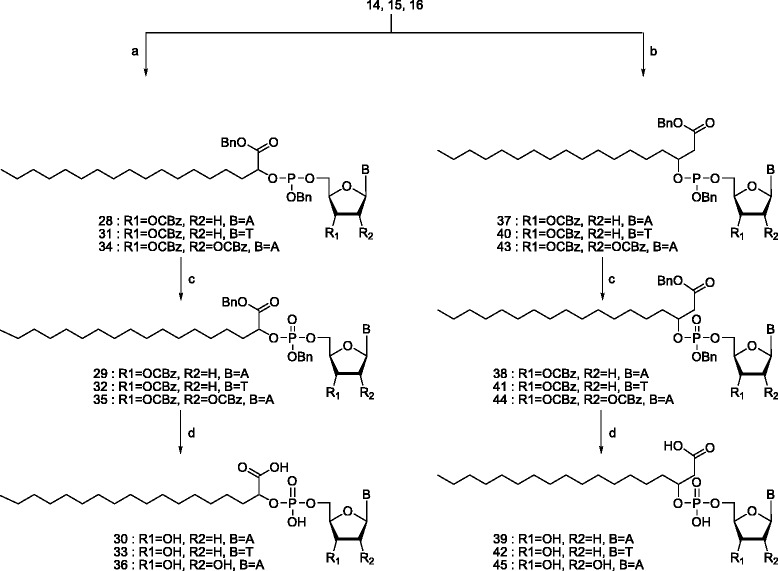
**Synthesis of the ribose and deoxyribose nucleolipids. a)** 12,1H-tetrazole, dry DCM, rt 4 h; **b)** 13, 1H-tetrazole, dry DCM, rt 4 h; **c)** H_2_O_2_, -78°C - rt, 30 min; **d)** H_2_, Pd/C, THF, K_2_CO_3_, H_2_O, rt 48 h, for 32 and 41, MeOH was used as co-solvent without adding K_2_CO_3_. 30, 36, 39 and 45 were isolated as 2 K^+^ salt.

The synthesis of the phosphotriesters was accomplished in a one-pot method by reacting **12** and **13** with the protected nucleosides **14**, **15**, **16** and **27** in the presence of 1H-tetrazole followed by oxidation with hydrogen peroxide at -78°C, resulting in the phosphotriesters **29**, **32**, **35**, **38**, **41**, **44**, **46**, and **48** (Scheme 7).

Deprotection of the benzyl and the carbobenzyloxy group of phosphotriesters **32** and **41** was performed by hydrogenolysis in the presence of Pd/C in THF/MeOH. Hydrogenolysis in THF/H_2_O as solvent in the presence of K_2_CO_3_, was carried out for the deprotection of the phosphotriesters of the adenine derivatives (**29**, **35**, **38**, **44**, **46**, and **48**) to obtain phosphodiesters **30**, **36**, **39**, **45**, **47**, **49**.

**Scheme 8:**
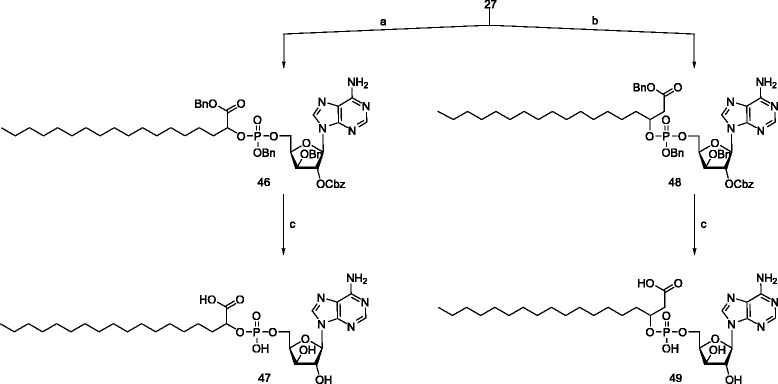
**Synthesis of xylose nucleolipids. a)** 12, dry DCM,1H-tetrazole, H_2_O_2_, rt, 4 h.; **b)** 13, dry DCM, 1H-tetrazole, H_2_O_2_, rt, 4 h.; **c)** H_2,_ Pd/C, THF, rt, 48 h. Compounds 47 and 48 were isolated as 2Na^+^ salt.

**Scheme 9:**
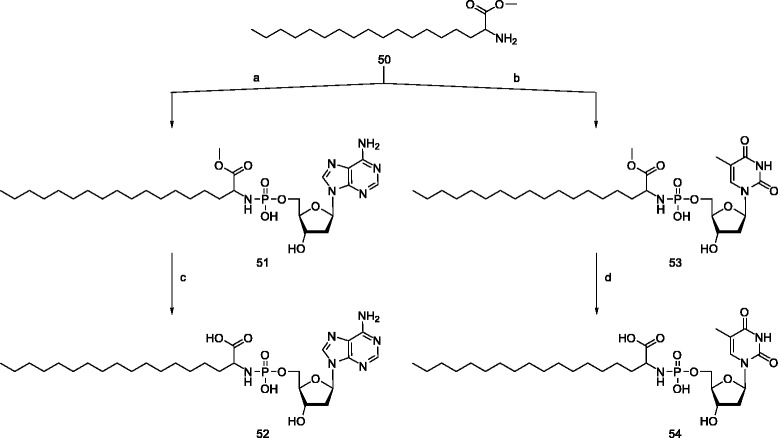
**Synthesis of the phosphoramidate nucleolipids. a)** dAMP, DCC, *t-*BuOH, H_2_O, reflux, 2 h; **b)** TMP, DCC, *t-*BuOH, H_2_O, reflux, 2 h; **c)** 0.5 N NaOH, MeOH, rt. Compounds 52 and 54 were isolated as 2Na ^+^ salt.

α-(±)-Aminostearic acid **50** was synthesized based on the method reported by Toth [37] and Porter [38], starting from 1-bromohexadecane and diethylacetamido malonate. The crude material was directly used for esterification to obtain compound **50** (Schemes 8 and 9).

The synthetic protocol, used for the synthesis of the phosphoramidates of dAMP **51** and dTMP **53** was based on literature prescription [39,40] using dicyclohexylcarbodiimide (DCC) as coupling agent for the conjugation of nucleotides and amino acid. The phosphoramidates **51**, **53** were obtained by refluxing the nucleoside monophosphates and the amino acid methyl ester in *t-*BuOH and water (5:1) in the presence of DCC (dicyclohexyldicarbodiimide). Deprotection of methyl ester was performed using 0.5 N NaOH in MeOH/H_2_O-5:1 at room temperature for 4 h result in the phosphoramidates **52** and **54**.

**Figure 2:**
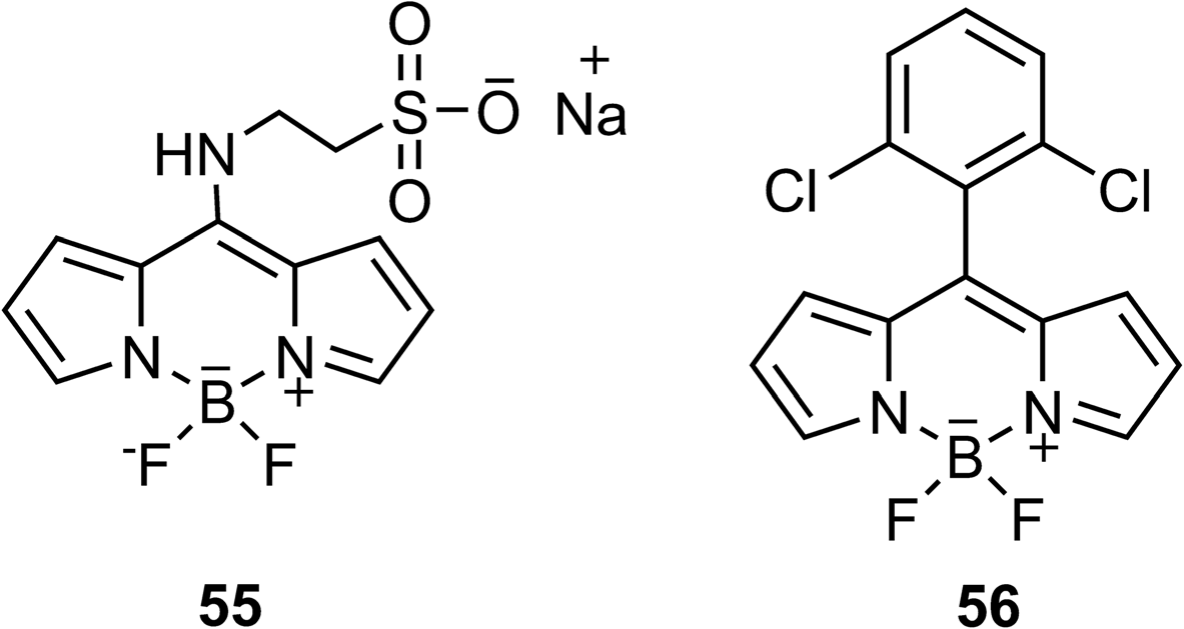
**Structures of fluorescent dyes used for the visualization of vesicles.**

The self-assembling properties of these nucleolipids were analyzed by fluorescent microscopy using water soluble, and organic soluble (chloroform) fluorescent dyes **55** and **56** respectively (Figure 2). Synthesis of the new, water-soluble BODIPY dye **55** was carried out by conjugating 8-S-Methyl BODIPY [41] with taurine in presence of sodium hydrogen carbonate in DMSO:DCM (1:1) at room temperature. BODIPY **56** was prepared according to the procedure previously reported by Dehaen [42,43] at rt.

The nucleolipids, which have been analyzed, consist of an α-hydroxy fatty acid (**30**, **33**, **36**, **47**), a β-hydroxy fatty acid (**39**, **42**, **45**, **49**) or a α-amino fatty acid (**52**, **54**). The polar head group is a nucleoside monophosphate (dAMP or dTMP) connected to the lipid by a phosphodiester (**30**, **33**, **36**, **39**, **42**, **45**, **47**, **49**) or by a phosphoramidate bond (**52**, **54**). The sugar is either a deoxyribose (**30**, **33**, **39**, **42**, **52**, **54**), or a ribofuranose (**36**, **45**) or a xylofuranose (**47**, **49**). The reason for this selection is that we would like to evaluate a) if the sugar moiety may influence the self-aggregation process, b) if oligomerization may lead to DNA and/or RNA sequences, c) if it would be possible to form 3′-O, 5′-O-cyclic phosphates in solution, d) if the properties of the leaving group may influence oligomerization or cyclic phosphate formation. For example, an α-hydroxy acid may lead to a 5-membered intermediate and a β-hydroxy acid to a 6-membered intermediate during activation of the phosphodiester bond (Scheme 1). A phosphoramidate may be activated as leaving group by acidification of the medium. A xylofuranose may lead easier to cyclic nucleoside formation than a ribofuranose. The availability of as well A as T nucleolipids would allow us to study mixed vesicles, in which aggregation may be influenced by base pairing.

For studying the aggregation of the compounds, following 2′-deoxynucleolipids have been used: **30**, **33**, **39**, **42**, **52**, and **54**. The fluorescent Bodipy dyes (**55**, **56**) were used to monitor self-assembly of the nucleolipids by visualization under fluorescent microscopy using a spin coat method on the surface of a microscopic glass plate. Vesicle formation of nucleolipids in water was facilitated by adding small amounts of organic solvents to solubilize respectively the dye (THF) or the nucleolipid (DMSO). Soon after dissolving the nucleolipid by vortexing, a structural transition towards thermodynamically more stable spherical structures is observed. Vesicular aggregations are formed ranging from about a few to 10 μm (large vesicles), depending on the dilution and solvent conditions. Also irregular (small and large tubular structures) aggregates are formed in some cases. A series of representative examples (most frequently occurring aggregates) for the phospholipids **30**, **33**, **42**, **52**, **54** are shown.

**Figure 3:**
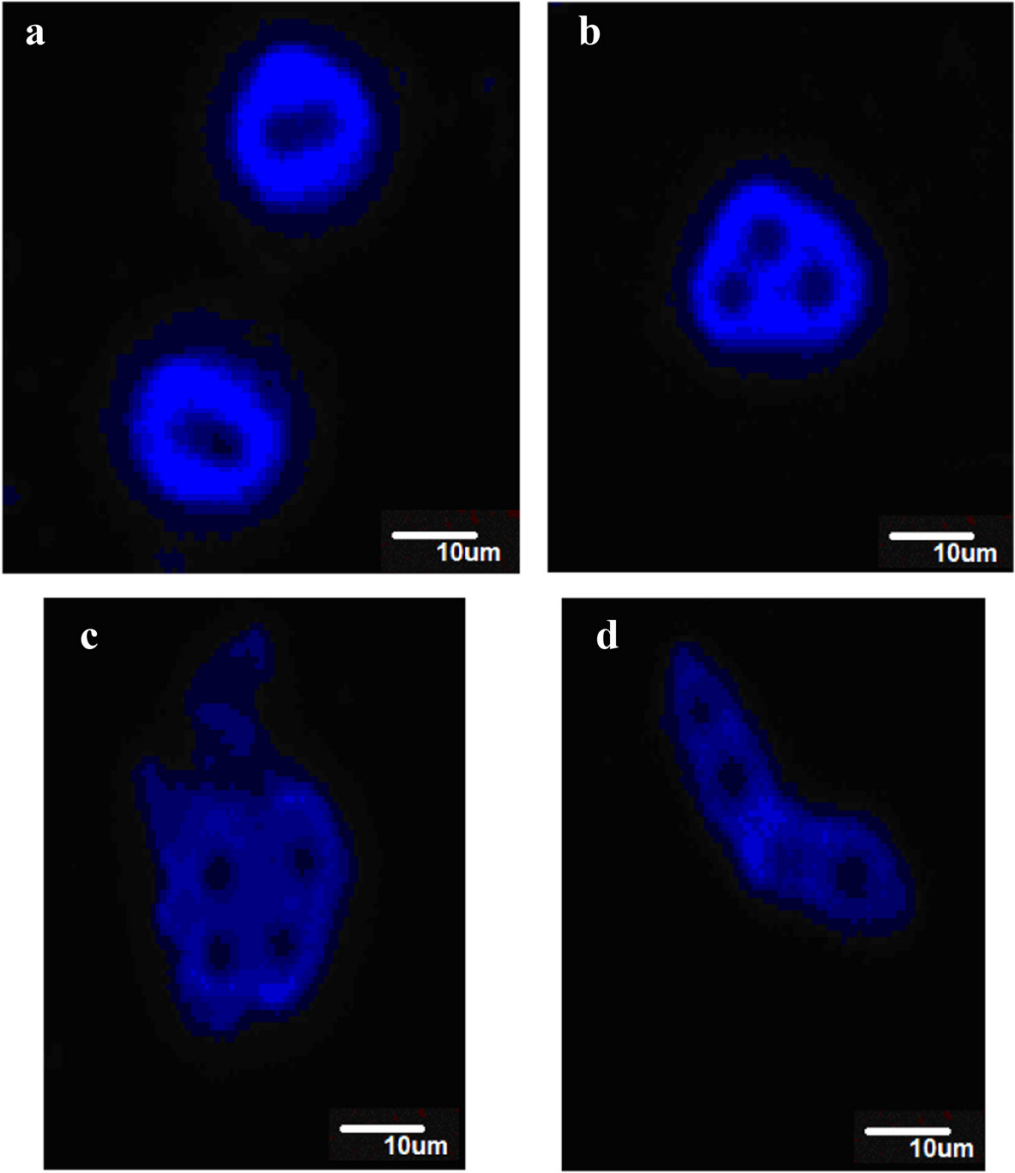
**Representative fluorescent images of compound 30 with dye 55.** Final concentrations of products for 3**a-b, d** =1.5 mM 30, 5.7 mM 55, 0.15 mM HCl, 7% DMSO in H_2_O; for 3**c** =1.1 mM 30, 4.4 mM 55, 0.11 mM HCl, 5% DMSO in H_2_O. (Scale bar 10 μm).

**Figure 4:**
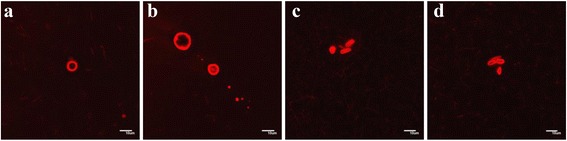
**Fluorescent microscopic images of the compound 33 with dye 56.** Final concentrations of products for 4**a-b** =0.4 mM 33, 0.013 mM 56 in H2O/dioxane/THF (3: 47:50); for 4**c-d** =0.8 mM 33, 0.025 mM 56, 6% THF in H_2_O. (Scale bar 10 μm).

Figure 3 shows microphotographs of compound **30** in H_2_O using dye **55** for visualization. Single vesicles are formed (Figure 3a) as well as several vesicle aggregates in which three (Figure 3b) or four (Figure 3c, d) water compartments are present. This could be intermediate stages in vesicle association or vesicle dissociation. Some of them (Figure 3d) are similar in morphology to the thread-like vesicles [7], which are formed before division in daughter vesicles. Figure 4 gives the images of compound **33** using dye **56**. Here, single, spherical vesicles are formed using water-miscible organic solvents (Figure 4a, b), in water vesicles tend to associate (Figure 4c, d).

**Figure 5:**
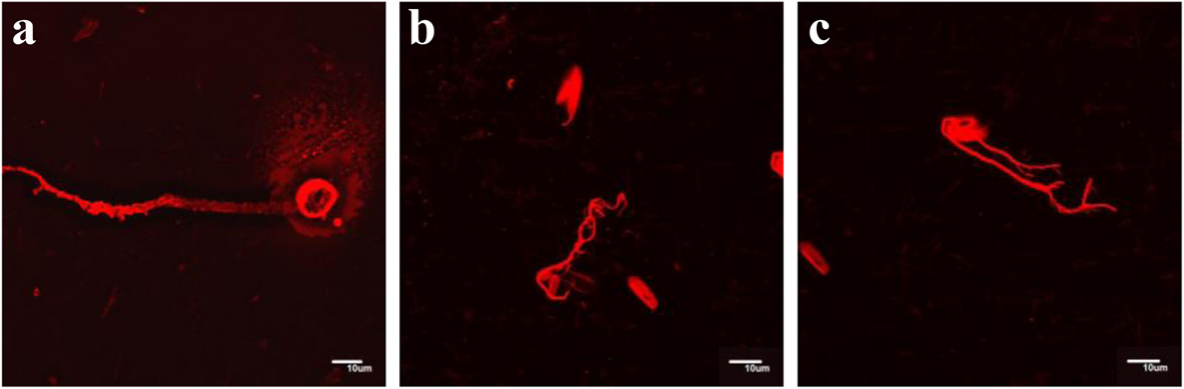
**Fluorescent microscopic images of the compound 42 with dye 56.** Final concentrations of products for 5**a-c** =0.3 mM 42, 0.009 mM 56, 5% THF in H_2_O. (Scale bar 10 μm).

**Figure 6:**
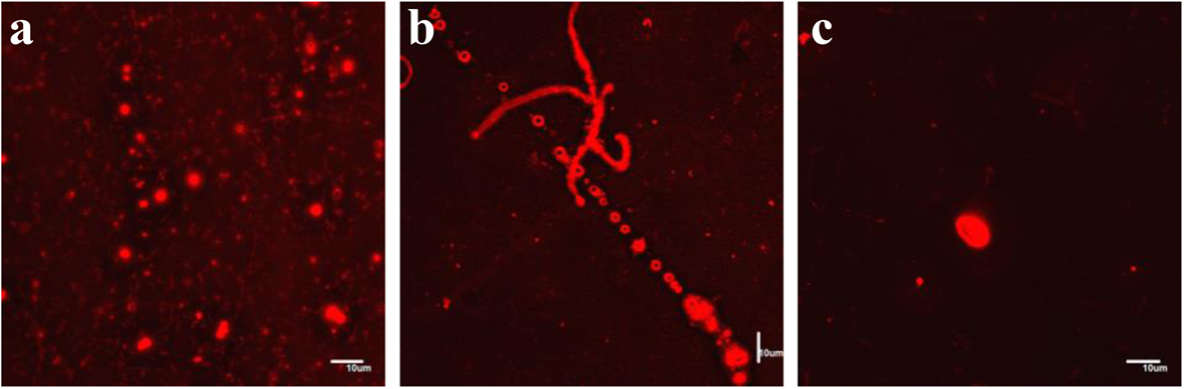
**Fluorescent microscopic images of the compound 52 with dye 56.** Final concentrations of products for 6**a-b** =0.39 mM 52, 0.013 mM 56 in H_2_O/dioxane/THF (3: 47:50); for 6**c** =0.66 mM 52, 0.022 mM 56, 5% THF in H_2_O. (Scale bar 10 μm).

**Figure 7:**
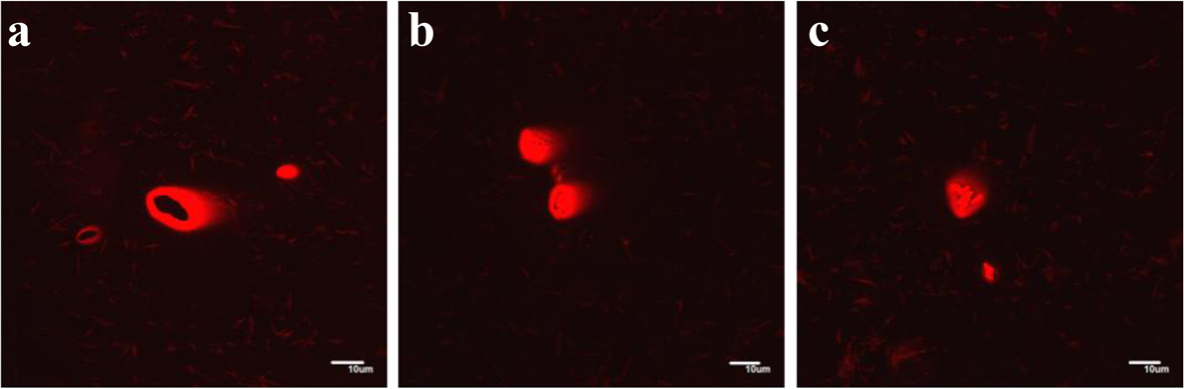
**Fluorescent microscopic images of the compound 54 with dye 56.** Final concentrations of products for 7**a-c** =0.3 mM 54, 0.009 mM 56, 2% THF in H_2_O. (Scale bar 10 μm).

Figure 5 is representative for the images observed using compound **42** and dye **56**. As well the vesicles (5a, b, c) are observed as rod-like structures (5a, c) which may be precursor structures for vesicle formation. Finally, the aggregates formed by the phosphoramidate conjugates with an adenine (**52**) and thymine (**54**) base moiety were visualized using dye **56**. The pictures of **52** (Figure 6 in THF/dioxane) and **54** (Figure 7b, c in water) shows the start of the formation of vesicle colonies [44].

For studying the potential of the nucleolipids to di(poly)merize and/or to form cyclic nucleotides, compounds with a deoxyribose (**30**, **33**, **39**, **42**, **52** and **54**), a ribose (**36**, **45**) or a xylose (**47**, **49**) sugar moiety were envisaged. Both basic and acid circumstances were considered. Oligomerization could occur by an intermolecular reaction in which the 2′-OH or 3′-OH group of the sugar moiety attacks the 5′-O-phosphoester function, using the hydroxy (amino) lipid as leaving group. The carboxylate group in the α- or β-position may catalyze this reaction. Alternatively, a 3′-O, 5′-O-cyclic nucleotide may be formed (by intramolecular reaction) which could oligomerize in solution. During the synthesis of the compounds, we already observed that some of them are not stable in acidic medium. Therefore, all compounds were treated in acid and in base medium (pH4 and pH12) for a period of 48 h. However in none of the cases, polymerization products were detected using NMR spectroscopy. These negative results could be explained by the high chemical stability of the nucleolipids (only starting material present) or by hydrolysis of the compounds in acidic and/or basic medium. Therefore, we have evaluated this stability for representative examples (**30**, **33**, **52**, **54**).

^31^P NMR was used to study the stability of the nucleolipids in acidic (pH4) and in base (pH12) environment. One-dimensional ^31^P spectra were used as a fast screening experiment to monitor degradation of the conjugates. Two-dimensional ^1^H-^31^P correlation spectra were used to characterize the ^31^P containing products formed by degradation of the nucleolipids. Correlations were established using a heteroTOCSY experiment with a DIPSI spinlock of 50 ms, allowing correlations of ^31^P resonances with several ^1^H resonances of adjacent spin systems.

The β-hydroxystearic acid containing nucleolipids (**39**, **42**, **45**, **49**) are stable in both acidic and basic conditions with no difference in the ^31^P and ^1^H NMR signals over time at different pH’s. All other compounds are stable in basic conditions (pH = 12 in D_2_O) while degradation occurs in acidic conditions. For phosphate diesters, a gel is formed instantly upon lowering pH in water. Although (hydro)gelation is an interesting property and promising application of nucleolipids, this was not further investigated [45]. Due to hampered NMR measurements in aqueous conditions, sample degradation in acid medium was monitored in DMSO.

**Figure 8:**
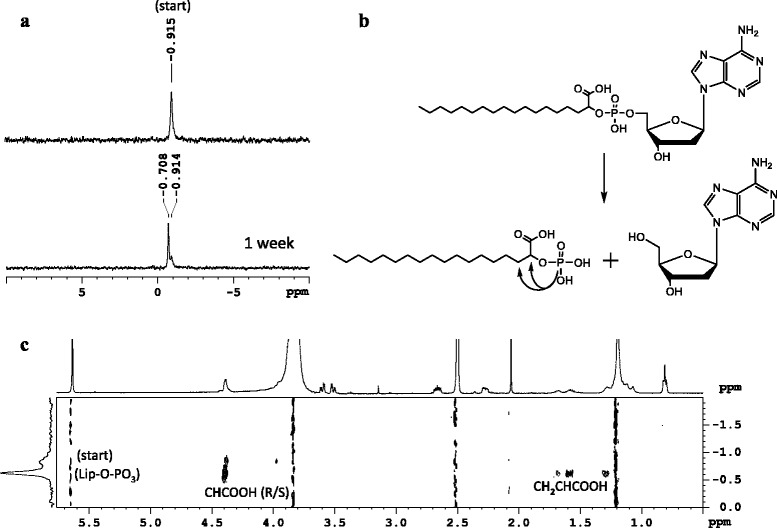
**NMR study of 30 in acid conditions (pH-5.22 in DMSO). (a)** time dependent degradation in acidic conditions monitored by 1D 31P NMR spectra. **(b)** degradation reaction of compound 30 in acidic conditions based on characterization of hydrolysis products by NMR. Arrows indicate cross peaks observed in the 2D spectrum depicted in C. **(c)** ^1^H-^31^P-heteroTOCSY after 1 day in acidic conditions.

**Figure 9:**
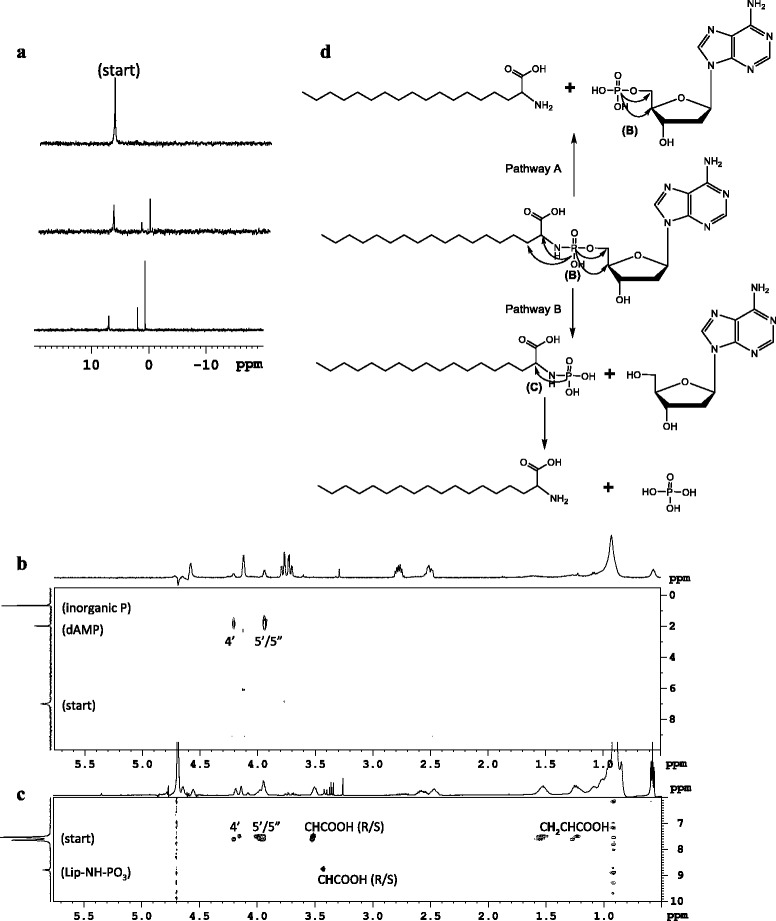
**NMR study of 52. (a)** time dependent degradation in D_2_O. **(b)** HP-heteroTOCSY after 1 day in D_2_O. **(c)** HP-heteroTOCSY after 1 day in DMSO. **(d)** degradation reaction of nucleolipid 52 in acidic conditions. Arrows indicate cross peaks observed in the 2D spectra depicted in **(b)** and **(c)**.

**Figure 10:**
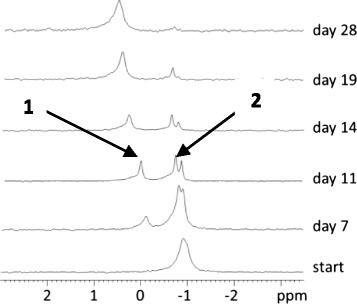
**Time dependent changes in 1D** ^**31**^ **P NMR spectrum of nucleolipid 33 in DMSO at acidic conditions.** Emergence of 2 degradation products coming from phosphate (1) and Lip-OPO_3_ (2).

An example NMR study on an nucleolipid diester with α-hydroxy stearic acid is given in Figures 8 and 9 for compound **30**. Original ^31^P signals appear in 1D ^31^P spectra between 0.1 and 0.0 ppm corresponding to Cα in R and S enantiomers in nucleolipid **30**. Due to degradation in acidic conditions, a new ^31^P signal rises slightly downfield (0.4 ppm) while the original signals decrease. In a 2D-heteroTOCSY the original signals close to 0 ppm correlate with protons in the spin systems of the ribose ring (5′/5″/4′) as well as the lipid (α, β, γ). The new signal at 0.4 ppm only correlates with protons of the lipid (α, β, γ), indicating that the covalent bond between lipid and phosphorus still exists after degradation. In acidic medium (pH4-5), the thymidine congener **33** is degraded in the same way as the adenine congener **30**, which shows that the cleavage mechanism is not dependent on the nucleobase (Figure 10).

Decomposition of the phosphoramidate **52** was first studied in aqueous, acidic conditions (Figure 10). We observed decrease of the original ^31^P signal (7 ppm) in D_2_O while to signals rose at 2 and 1 ppm. The latter were assigned to dAMP and inorganic phosphate respectively using [^1^H,^31^P]-heteroTOCSY. Since the ratio of both new signals is constant over time, we suggest that initial cleavage occurs at both amide and ester bonds in the P linkage yielding dAMP and dA respectively. While dAMP is stable in the reaction medium, the phosphorylated lipid rapidly undergoes hydrolysis releasing inorganic phosphate. In DMSO, stability of the phosphorylated lipid is increased, making it observable as an intermediate in 1D and 2D NMR spectra (^31^P signal at 8.8 ppm). The H8 signals from the nucleobases in **52**, dAMP and dA are nicely resolved and allowed to determine a ratio of 1/4 for dAMP and dA formation:20% of adenosine monophosphate and lipid are formed via pathway A and 80% of deoxyadenosine, inorganic phosphate and lipid are formed in pathway B. The thymine containing congener of **52** (**54**) also follows both degradation pathways in acidic conditions: 53% pathway A with formation of dTMP and lipid and 47% pathway B with formation of thymidine, inorganic phosphate and the lipid. Indicating that the nucleobase influences the ratio between P-O and P-N bond cleavage.

To summarize, among all investigated systems, only α-amino compounds have shown the desired, however nucleobase dependent, bond breakage upon acidifying; the only problem being that the nucleophile is water and not the 2’- or 3’- hydroxyl groups. The β-hydroxystearic acid containing nucleolipids are stable in both acidic and basic conditions. This difference between α and β derivatives is analogues to previous calculations (done on a model in which the nucleoside had been replaced by a methyl group), showing that the formation of a six-membered intermediate by the attack of the β-carboxyl group is higher in energy (5 kcalmol-1) than the five-membered ring formed by an α-carboxyl group [22]. The proposed mechanism, predicting acidic instability, in Scheme 1b is supported by the fact that only amino derivatives cleaved at the desired bond, due to the preferred protonation of a phosphoramidate over a phosphodiester.

## Experimental

### Benzyl 2-hydroxyoctadecanoate (4)

To a solution of α-hydroxy stearic acid **3** (10 g, 33.28 mmol), triethylamine (6.73 g, 9.25 ml, 66.56 mmol), TBAI (1.23 g, 3.33 mmol) in toluene (150 mL), benzyl bromide (5.7 g, 5.03 mL, 33.3 mmol) is added, and held at 90°C overnight. After the completion of the reaction, the reaction mixture is triturated with diethyl ether, the precipitate formed was washed with ether and dried to obtain the desired compound **4** as white solid (7.25 g, 55.7%). ^1^H NMR (300 MHz, CDCl3, ppm): *δ* 7.37 (m, 5H, Ar), 5.25 (q, 2H, CH_2_), 4.23 (m, 1H), 2.79 (br, 1H, -OH), 1.82-1.73 (m, 1H), 1.69 (m, 1H), 1.25 (s, 28H, -CH_2_), 0.90 (t, -CH_2_). ^13^C NMR (300 MHz, CDCl3, ppm): *δ* 175.41(C = O), 135.37(-C-), 128.75 (-C-Ar), 128.65 (-C-Ar), 128.44 (-C-Ar), 70.64 (-C-H), 67.36 (Ar-CH_2_), 34.52 (-CH_2_), 32.05 (-CH_2_), 29.82 (-CH_2_), 29.79 (-CH_2_), 29.66 (-CH_2_), 29.58 (-CH_2_), 29.49 (-CH_2_), 24.78 (-CH_2_), 22.81(-CH_2_), 14.24 (-CH_3_). HRMS (ESI+) m/z Calculated for C_25_H_43_O_3_ (MH+): 391.3212 found 391.2869. C_25_H_42_O_3_Na (MNa+): 413.3031 found 413.2918.

### Benzyl 3-hydroxy stearate (9)

To a solution of β-hydroxy stearic acid **8** (2 g, 6.65 mmol), triethylamine (1.2 ml, 8.65 mmol), and TBAI (0.25 g, 0.66 mmol) in toluene, benzyl bromide (1.14 g, 0.8 mL, 6.65 mmol) was added, and stirred at 90°C overnight. After the completion of the reaction, the reaction mixture is triturated with diethyl ether, and the precipitate formed was washed with diethyl ether (2 × 50 mL). The residue obtained was purified on silica gel column chromatography using dichloromethane to obtain the desired compound **9** [46] as white solid (1.47 g, 56%). ^1^H NMR (300 MHz, CDCl_3_): 7.34(m, 5H-Ph), 5.14 (s, 2H, CH_2_-Ph), 4.01 (m, 1H), 2.9 (br s, 1H, OH), 2.58-2.41 (m, 2H, CH_2_), 1.43(m, 2H, CH_2_), 1.41(m, 2H, CH_2_), 1.25 (s, 24H, CH_2_), 0.9 (t, 3H, CH_3_). ^13^C (300 MHz, CDCl_3_):172.50 (C = O), 135.32(C-Ph), 128.28(C-Ph), 128.02(C-Ph),127.92(C-Ph), 67.73(C, C-OH), 66.12(C, CH_2_-Ph), 41.08 (C, CH_2_),36.24 (C, CH_2_), 31.60 (C, CH_2_), 29.37 (C, CH_2_), 29.34 (C, CH_2_), 29.26 (C, CH_2_), 29.24 (C, CH_2_), 29.20 (C, CH_2_), 29.03 (C, CH_2_), 25.14 (C, CH_2_), 22.36 (C, CH_2_), 13.78 (C, CH_3_). HRMS(ES+) m/z Calculated for C_25_H_42_O_3_ (MH+): 391.3206, found 391.3199.

### Benzyl, (benzyl stearate)-2-yl, *N*, *N*-diisopropyl phosphoramidite [(±)-12]

To a stirring mixture of benzyloxy bis(N, N’-diisopropylamino)phosphine (10 g, 29.55 mmol) and alpha-hydroxy benzyl stearate (5.77 g, 14.77 mmol) in dry DCM (100 mL), was added 0.5 M solution of 1H-Tetrazole (29.6 mL, 14.77 mmol) in ACN dropwise at 0°C. After the addition, cooling bath was removed and the reaction mixture was allowed to stir at rt for 15 min. The reaction mixture was diluted with DCM (200 mL), and the organic layer was washed with 1 M TEAB solution. The extracts were dried over Na_2_SO_4_ and concentrated in vacuum and purified by flash column chromatography using eluent 400:50:10-Hexane:EtOAc:TEA (R*f =* 0.5) to obtain the phosphoramidite **12** (7.10 g, 71%). P^31^ NMR (CDCl_3_): δ 149.10, 148.69.

### Benzyl, (benzyl stearate)-3-yl, N, N-diisopropyl phosphoramidite [(±)-13]

To a stirring mixture of benzyloxy Bis(N, N’-diisopropylamino) phosphine (**11**, 1.74 g, 5.12 mmol) and β-hydroxy stearic benzyl ester (**9**, 1 g, 2.56 mmol) in dry DCM (10 mL) was added 0.5 M solution of 1H-tetrazole (5.12 mL, 2.56 mmol) in ACN dropwise at 0°C. After the addition, cooling bath was removed and the reaction mixture was allowed to stir at room temperature for 15 min. The reaction mixture was diluted with DCM (150 mL), and was washed with 1 M TEAB (100 mL) solution. Dichloromethane layer was dried over Na_2_SO_4_ and concentrated *in vacuo*, and the obtained oil was purified by silica gel flash column chromatography using the eluent 400:50:10-Hexane:EtOAc:TEA (R*f* = 0.5) to get **13** as an pale yellow oil (0.985 g, 61%). ^31^P NMR (300 MHz, CDCl_3_, ppm): δ 147.63, 147.07.

### 3-O-benzyl-1,2:5,6-Di-O-isopropylidene-α-D-glucofuranose (17)

To a stirring solution of diacetone glucose (10 g, 38.42 mmol) in anhydrous DMF at 0°C, was added sodium hydride (60% in mineral oil (w/w), total 2.3 g, 57.63 mmol) portion wise. Stirring was continued for 1 h at 0°C, and then benzyl bromide (5.51 g, 46.10 mmol) was added drop wise. After addition, ice bath was removed, and stirring continued overnight at room temperature. After completion of the reaction, excess of sodium hydride in the reaction mixture was quenched by the addition of ice cold water (25 mL). The reaction mixture was extracted with EtOAc (4 × 150 mL) and the combined organic phase was dried (Na_2_SO_4_) and concentrated under vacuum, and the residue was purified by silica gel column chromatography using eluents hexane:ethylacetate-8:2 to give the title compound **17** (13 g, 96%) as oil. ^1^H NMR (300 MHz, CDCl_3_, ppm): 7.33-7.28 (m, 5H, Ar), 5.88 (d, 1H, 1′-H), 4.68-4.62 (m, 2H, CH_2_-Ar), 4.56 (d, 1H, 2′-H), 4.37-4.33 (m, 1H, 5′-H), 4.16-4.06 (m, 2H, 6′-H, 6″-H), 4.01-3.96 (m, 2H, 3′-H, 4′-H), 1.47 (s, 3H, CH_3_), 1.41(s, 3H, CH_3_), 1.35(s, 3H, CH_3_), 1.28(s, 3H, CH_3_). ^13^C NMR (300 MHz, CDCl_3_, ppm): δ 137.66, 128.33, 127.76, 127.58, 111.65, 108.86, 108.86, 105.27, 82.64, 81.68, 81.31, 72.50, 72.29, 67.35, 26.81, 26.75, 26.20, 25.40.

### 3-*O*-benzyl-1,2-*O*-isopropylidene-α-D-glucofuranose (18)

In a 250 mL round bottom flask 3-O-benzyl-1,2:5,6-di-O-isopropylidene-α-D-glucofuranose (7.00 g, 19.97 mmol) was dissolved in 1:1 methanol/1% aqueous sulfuric acid (110 mL), and the resulting solution stirred at rt. After completion of the reaction (approximately 5 h, monitored by TLC), the reaction mixture was quenched with triethylamine (pH7). The residue was concentrated *in vacuo* to afford the crude residue as syrup, which was purified by silica gel flash column chromatography using 5% MeOH in dichloromethane, affording the title compound **18** [33] (5.05 g, 81%) as a colorless solid. ^1^H NMR (300 MHz, CDCl_3_, ppm): 7.31-7.28 (m, 5H, Ar), 5.91 (m, 1H, 1′-H), 4.71 (dd, 2H, CH_2_-Ar), 4.51 (s, 1H), 4.13 (m, 2H), 4.09 (m, 1H), 3.81 (dd, 2H), 3.03 (br, 1H), 2.02, (s, 1H), 1.47 (s, 3H), 1.29 (s, 3H). ^13^C NMR (300 MHz, CDCl_3_, ppm): δ171.29, 137.41, 128.70, 128.64, 128.11, 127.81, 111.80, 105.16, 82.21, 81.97, 80.01, 69.11, 64.33, 60.45, 26.73, 26.22, 21.03, 14.19. HRMS (ESI+) m/z Calculated for C_16_H_22_O_6_ (MH+): 311.1489, found 311.1486.

### 3-*O*-benzyl-1,2-*O*-isopropylidene-β-D-xylofuranose (20)

To a stirring solution of 3-O-benzyl-1,2- isopropylidene-α-D-glucofuranose (5 g, 16.11 mmol) in water (50 mL) was added sodium meta-periodate (4.13 g, 19.33) at room temperature. Stirring continued until consumption of starting material. The reaction mixture was diluted with ethanol (80 mL) and stirring continued for another 30 min. The reaction mixture is filtered on celite and the celite pad washed with ethanol. The filtrate was transferred to a 500 mL round bottom flask, and cooled to 0°C. Sodium borohydride (0.67 g, 17.72 mmol) was added in small portions to the filtrate. After completion of the addition, ice bath was removed and stirring continued for 2 h at room temperature. The reaction mixture was neutralized by drop wise addition of acetic acid, concentrated *in vacuo* and the residue is purified by silica gel column chromatography to get the title compound **20** [33,47] (4 g, 88.5%). ^1^H NMR (300 MHz, CDCl_3_, ppm): δ 7.37-7.28 (m, 5H, Ar-H), 5.99 (d, 1H, 1′-H), 4.73 (dd, CH_2_-Ar), 4.64 (s, 1H, 4-′H), 4.30 (dd, 1H, 2′-H), 3.92 (d, 1H, 3′-H), 3.91-3.1 (m, 2H, 5′-H, 5″-H), 2.24 (dd, 1H), 1.48 (s, 3H, CH_3_), 1.33 (s, 3H, CH_3_). ^13^C NMR (300 MHz, CDCl_3_, ppm): δ 137.19 (-C-, Ar), 128.78(CH, Ar), 128.31 (CH, Ar), 127.85 (CH, Ar), 111.90 (C), 105.20(C-1′), 82.87 (C-4′), 82.60 (C-2′), 80.22 (C-3′), 72.04 (CH_2_-Ar), 61.09 (C-5′), 26.93 (CH_3_), 26.43(CH_3_). HRMS (ESI+) m/z Calculated for C_15_H_20_O_5_ [M + Na]+ : 303.1203, found 303.1207.

### 3-*O*-benzyl-1,2-*O*-isopropylidene-β-D-xylofuranose (21)

To a suspension of 3-O-Benzyl-1,2-O-isopropylidene-β-D-xylofuranose (4 g, 14.26 mmol) in benzoyl chloride (1.64 g, 2 mL,17.12 mmol), was added sulfamic acid (0.55 g, 5.70 mmol). The reaction mixture was stirred at 60°C for 3 h, allowed to come to room temperature and the reaction mixture was poured into ice cold saturated NaHCO_3_ solution, and extracted with minimum amount of diethyl ether. The organic solvents were dried on Na_2_SO_4_ and concentrated under reduced pressure. The residue was purified by silica gel column chromatography using eluents hexane:ethyl acetate (8:2) to obtain the compound **21** [36] (4.8 g, 91%). ^1^H NMR (300 MHz, CDCl_3_, ppm): δ 8.01 (d, 2H), 7.54-7.51 (m, 1H), 7.40-7.37 (m, 2H), 7.30-7.26 (m, 4H), 7.24 (d, 1H), 5.99 (d, 1H), 4.70-4.60 (m, 3H), 4.57-4.48 (m, 3H), 4.04 (br s, 1H), 1.49 (s, 3H, CH_3_), 1.32 (s, 3H, CH_3_). ^13^C NMR (300 MHz, CDCl_3_, ppm): δ166.21, 137.16, 133.03, 129.88, 129.75, 128.53, 128.31, 128.07, 127.77, 111.82, 105.32, 82.13, 81.52, 78.14, 71.88, 26.83, 26.25.

### (9-(2′-*O*-acetyl-3′-*O*-benzyl-5′-*O*-benzoyl) β-D-xylofuranosyl) 6-*N*-benzoyladenine (23)

To a cooled solution of 5-O-benzoyl-3-O-benzyl-1,2-O-isopropylidene β-D-xylofuranose (4 g, 10.40 mmol) in AcOH (40 mL) and Ac2O (15 mL) was added drop wise sulfuric acid (1.5 mL). Stirring was continued at room temperature until TLC analysis shows disappearance of the starting material, the mixture was poured into ice-water, extracted with CHCl_3_ (3 × 100mL), washed with saturated NaHCO_3_ (200 mL) and dried over Na_2_SO_4_. The solvent was removed under reduced pressure and the residue is used directly in the next step. TLC-(7:3 hexanes–EtOAc-Rf = 0.4).

To a suspension of **22** (5 g, 11.67 mmol) and N^6^-benzoyladenine (4.2 g, 17.50 mmol) in anhydrous acetonitrile (60 mL) was added drop wise 1 M SnCl_4_ in dichloromethane (23.5 mL, 23.34 mmol) under argon. The resulting mixture was allowed to stir for 4 h at room temperature. After completion of the reaction, saturated aq NaHCO_3_ was added slowly until the evolution of carbon dioxide ceased. Then the mixture was filtered through a pad of Celite 545, that was subsequently washed with CHCl_3_ (3x100 mL). The combined filtrate was washed successively with saturated aq NaHCO_3_ (3 × 100 mL) and brine (2x100 mL), dried (Na2SO_4_). The filtrates were concentrated under reduced pressure. The residue obtained was purified by silica gel column chromatography using 3%MeOH in CHCl_3_ as eluent (Rf-0.5), affording nucleoside **23** [36] (6.12 g, 86%) as a colorless solid. ^1^H NMR (500 MHz, DMSO- DMSO-d_6_, ppm): δ 9.36 (br s, 1H, -NH), 8.77 (s, 1H, H-2), 8.42 (s, 1H, H-8), 8.03 (dd, 5H, Ar), 7.59 (q, 2H, Ar), 7.50 (t, 2H, Ar), 7.43 (t, 2H, Ar), 7.27 (m, 5H, Ar), 6.44 (s, 1H, H-1′), 5.54 (s, 1H, H-2′), 4.76 (dd, 2H), 4.74 (dd, 1H), 4.65 (m, 1H), 4.16 (d, 1H),2.18 (s, 3H, CH_3_). ^13^C NMR (500 MHz, DMSO- d_6_, ppm): δ 169.69 (C = O, CH_3_), 166.38 (C = O, Ar), 164.63 (C = O, Ar-NH), 153.03 (C-2), 151.57 (C-6), 149.58 (C-4), 141.81 (C-8), 136.24 (CH-Ar), 133.84 (CH-Ar), 133.45 (CH-Ar), 132.92 (CH-Ar), 129.88 (CH-Ar), 129.68 (CH-Ar), 129.02 (CH-Ar), 128.84 (CH-Ar), 128.59 (CH-Ar), 128.39 (CH-Ar), 127.99 (CH-Ar), 122.92 (CH-Ar), 87.82(1′-C), 81.18 (4′-C), 79.98 (3′-C), 79.75 (C-2′), 72.36 (CH_2_-Ar), 62.43 (C-5′), 20.99 (CH_3_). HRMS (ESI+) m/z Calculated for C_33_H_29_N_5_O_7_ (MH+): 608.2139, found 608.2135.

### 9-(3′-O-benzyl) β-D-xylofuranosyl)-adenine (24)

To a solution of nucleoside **23** (2 g, 3.29 mmol) in MeOH (20 mL) was added saturated methanolic ammonia (100 mL) in a sealed tube and the mixture was stirred at 85°C for 3 h. After completion of the reaction, the reaction mixture was concentrated to dryness under reduced pressure and the residue was coevaporated with toluene (5x10 mL). The residue obtained was purified by column chromatography using 5% MeOH in CHCl_3_ to afford nucleoside **24** (0.8 g, 68%). ^1^H NMR (500 MHz, DMSO-d_6_, ppm): δ 8.17 (s, 1H, H-2), 8.14 (s, 1H, H-8), 7.35-7.27 (m, 7H, Ar, -NH_2_), 6.04 (d, 1H, 2′-OH), 5.95 (s, 1H, 1′-H), 4.94 (br, 2H, 5′-OH), 4.68 (s, 1H, 2′-H), 4.67 (dd, 2H, CH_2_), 4.34 (m, 1H, 4′-H), 4.05 (br s, 1H, 3′-H), 3.81-3.73 (m, 5′-H, 5″-H). ^13^C NMR (500 MHz, DMSO- DMSO-d_6_, ppm): δ 156.04 (C-6), 152.67(C-2), 149.21 (C-4), 139.01 (C-8), 138.04(-C-, Ar), 128.29(-CH, Ar), 127.56 (-CH, Ar), 127.40 (-CH, Ar), 118.67(C-5), 88.74 (1′-C), 82.68 (3′-C), 77.26 (2′-C), 71.29 (CH_2_-Ar), 59.44 (5′-C). HRMS (ESI+) m/z Calculated for C_17_H_19_N_5_O_4_ (MH+): 358.1509, found 358.1510.

### 9-(5′-*O*-tert-butyldimethylsilyl-3′-*O*-benzyl) β-D-xylofuranosyl) adenine (25)

To a cooled suspension of 9-(5′-O-tert-butyldimethylsilyl-3-*O*-Benzyl-β-D-xylofuranosyl)-adenine (6 g, 16.78 mmol) and imidazole (2.85 g, 41.97 mmol) in anhydrous N, N- dimethylformamide was added tert-butyldimethylchlorosilane (3.03 g, 16.78 mmol) in anhydrous DMF under argon. The reaction mixture was allowed to stir for 20 h at room temperature. After completion of the reaction, the organic solvent was removed under high vacuum. The residue was dissolved in 150 mL of ethyl acetate, the solution was washed with two times 80 mL of water, and one time with 30 mL of brine and the extract was dried on Na_2_SO_4_ and the organic solvent was concentrated under reduced pressure. The residue obtained was purified on silica gel column chromatography using 2% MeOH in DCM to obtain the compound **25** as colorless solid (7.56 g, 95%). ^1^H NMR (500 MHz, CDCl_3_, ppm): δ 8.16 (s, 1H, H-2), 8.11 (s, 1H, H-8), 7.33 (m, 7H, Ar, -NH_2_), 6.05 (d, 1H, 2′-OH), 5.96 (d, 1H, 1′-H), 4.69 (s, 2H, 2′-H), 4.66-4.53 (m, Ar-CH_2_), 4.32 (m, 1H, 4′-H), 4.06 (m, 1H, 3′-H), 4.00-3.86 (m, 2H, 5′-H, 5″-H), 0.85 (s, 9H, -(CH_3_)_3_), 0.02 (s, 6H, -(CH_3_)_2_). ^13^C NMR (500 MHz, DMSO-d_6_, ppm): δ 155.85(C-6), 153.79 (-C = O, Cbz) 153.16(C-2), 149.52 (C-4), 139.04(C-8), 136.70 (-C-), 134.41 (-C-), 128.83 (-CH-, Ar), 128.68 (-CH-, Ar), 128.53 (-CH-, Ar), 128.50, (-CH-, Ar) 128.17 (-CH-, Ar), 127.91 (-CH-, Ar), 119.30 (C-5), 86.89 (C-1′), 83.31 (C-4′), 82.91 (C-3′), 79.92 (C-2′), 72.45 (-CH2, Bn), 70.57 (-CH2, Cbz), 60.37 (C-5′), 25.88 (-(CH_3_)_3_), 18.29 (-C-), -5.31 (CH_3_), -5.42 (CH_3_). HRMS (ESI+) m/z Calculated for C_23_H_33_N_5_O_4_Si_1_ (MH+): 472.2374, found: 472.2379.

### (9-(5′-*O*-tert-butyldimethylsilyl-3′-*O*-benzyl-2′-*O*-benzyloxycarbonyl) β-D-xylofuranosyl) adenine (26)

To a solution of 9-(5′-*O*-tert-butyldimethylsilyl-3′-*O*-Benzyl-2′-*O*-benzyloxycarbonyl-β-D-xylofuranosyl)-adenine (2.5 g, 53 mmol) and DMAP (1.3 g, 10.60 mmol) in 50 mL of anhydrous dichloromethane was added benzyl chloroformate (CbzCl, 1.8 g, 1.78 mL, 10.60 mmol) at 0°C under argon atmosphere. After stirring for 72 hours at room temperature, the reaction mixture was diluted with DCM (200 mL) and washed with cold 1.0 M HCl aqueous solution (50 mL) and then with water (100 mL). The organic layer was dried over anhydrous NaSO_4_, filtered, and concentrated under reduced pressure. The residue obtained, was purified by silica gel column chromatography (hexane/EtOAc-10:1 to 1:1 v/v) to obtain **26** (2.85 g, 89%). ^1^H NMR (500 MHz, CDCl3, ppm): δ 8.25 (s, 1H, H-2), 8.05 (s, 1H, H-8), 7.28-7.17 (m, 10H, Ar), 6.38 (br, m, 2H, -NH_2_), 6.29 (s, 1H, 1′-H), 5.11 (s, 2H, CH_2_-Cbz) 4.64 (s, 2H, 2′-H), 4.61-4.52 (m, Ar-CH_2_), 4.27 (m, 1H, 4′-H), 4.10 (d, 1H, 3′-H), 3.97-3.87 (m, 2H, 5′-H, 5″-H), 0.82 (s, 9H, -(CH_3_)_3_), 0.01 (s, 6H, -(CH_3_)_2_). ^13^C NMR (500 MHz, CDCl3, ppm): 155.85, 153.79, 153.16, 149.52, 139.04, 136.70, 134.41, 128.83, 128.68, 128.53, 128.50, 128.17, 127.91, 119.30, 86.89, 83.31, 82.91, 79.92, 72.45, 70.57, 60.37, 25.88, 18.29, -5.31, -5.42. HRMS (ESI+) m/z Calculated for C_31_H_39_N_5_O_6_Si_1_ (MH+): 606.2742, found: 606.2729.

### (9-(3′-O-benzyl-2′-*O*-benzyloxycarbonyl) β-D-xylofuranosyl) adenine (27)

To a solution of **26** (2 g, 33 mmol) in 30 mL of anhydrous THF was added 1 M TBAF in THF (8.63 g, 330 mmol) at 0°C under N_2_ atmosphere. The solution was stirred for 12 h at room temperature, and all volatiles were removed using a rotary evaporator. The residue was dissolved in EtOAc (100 mL) and washed with cold saturated NaHCO_3_ solution (30 mLx2), and brine (30 mL). The organic solvent was dried over Na_2_SO_4_ and filtered. The filtrate was concentrated and the obtained residue was purified by silica gel column chromatography (CH2Cl_2_/MeOH-9:1) to give **27** (1.41 g) as white solid in 86% yield. ^1^H NMR (500 MHz, CDCl_3_, ppm): δ 8.15 (s, 1H, H-2), 8.14 (s, 1H, H-8), 7.37-7.28 (m, 10H, 2xAr), 6.20 (s, 1H, 1′-H), 5.71 (t, 1H, 2′-H), 5.17 (s, 2H, -NH2), 5.03 (t, 1H, 5′-OH), 4.75 (dd, 2H, CH_2_-Ar), 4.38 (m, 1H, 4′-H), 4.30 (m, 1H, 3′-H), 3.78 (m, 2H, 5′-H, 5″-H). ^13^C NMR (500 MHz, CDCl_3_, ppm): 156.05, 153.24, 152.81, 149.14, 138.68, 137.57, 134.96, 128.53, 128.51, 128.32, 128.31, 127.70, 127.53, 118.52, 85.67, 82.58, 81.98, 79.90, 71.46, 69.75, 59.07. HRMS (ESI+) m/z Calculated for C_25_H_25_N_5_O_6_ (MH+):492.1877, found: 492.1881.

### 3′-*O*-benzyloxycarbonyl-2′-deoxyadenosine-5′-*(O*-(benzyl stearate)-2-yl, *O*-benzyl) phosphate (29)

To a stirred solution of **14** (1 g, 25.94 mmol) and **12** (2.45 g, 38.92 mmol) in 5 mL dry DCM was added 0.5 M solution of 1H-Tetrazole (25.94 mL, 129.74 mmol) drop wise at 0°C. The reaction mixture was allowed to stir at room temperature for 4 h. Then the reaction mixture was cooled down to -78°C and hydrogen peroxide 35% (W/V, 10 mL) was added. After stirring for 5 min at -78°C, cooling bath was removed and the reaction mixture was allowed to stir at room temperature for further 30 min. The reaction mixture is diluted with DCM (150 mL) and washed with 1 M phosphoric acid (70 mL), 5% aq. sodium bicarbonate (70 mL) and with brine (60 mL), dried over sodium sulfate, filtered and concentrated *in vacuo*. The obtained residue was purified by column chromatography using EtOAc to obtain the title compound **29** as oil. ^1^H NMR (300 MHz, DMSO-d_6_, ppm): δ 8.33 (m, 1H, H-2), 8.18 (m, 1H, H-8), 7.38 -7.26 (m, 15H, Ar-H), 5.64 (br s, 1H), 5.34-5.04 (m, 6H, 3xCH_2_), 4.90-4.82 (m, 1H), 4.32 (m, 2H), 2.17 (s, 1H), 1.78 (m, 2H), 1.25 (m, 28H, CH_2_), 0.89 (t, 3H, CH_3_). ^13^C NMR (500 MHz, DMSO-d6, ppm): δ 170.16, 155.59, 154.37, 153.28, 139.08, 138.93, 134.83, 128.85, 128.72, 128.55, 128.18, 128.13, 128.01, 127.97, 120.04, 84.20, 83.03, 82.92, 70.31, 67.40, 37.55, 33.09, 32.06, 29.84, 29.79, 29.65, 29.49, 19.16, 24.66, 22.82, 14.24. HRMS (ESI+) m/z Calculated for C_50_H_67_N_5_O_10_P_1_ (MH+): 928.4619, found 928.4582.

### 2′-deoxyadenosine-5′-(*O*-stearic acid-3-yl) phosphate (30)

To the solution of **29** (0.2 g, 0.21 mmol) in THF, K_2_CO_3_ (60 mg, 4.31 mmol) and water (2 mL) is added, followed by Palladium (10%) on charcoal, and stirring continued at room temperature under Hydrogen for 72 h. After completion of the reaction, the mixture is filtered on celite 545, and the celite pad was washed with THF:Water-1:1, and the filtrate was evaporated and purified by column chromatography using eluent DCM:MeOH:H_2_O-17:7:1 to obtain the potassium salt of **30** as white solid. ^1^H NMR (500 MHz, DMSO-d_6_, ppm): δ 8.37 (d, 1H, H-2), 8.12 (d, 1H, H-8), 7.25 (t, 2H), 6.37 (m, 1H), 5.43 (br s, 1H), 4.44 (br, 1H) 4.29 (m, 1H), 3.96 (t, 1H), 3.90-3.85 (m, 1H), 3.78 (m, 1H), 2.74 (m, 1H), 2.28 (m, 1H), 1.66 (m, 1H), 1.511 (m, 1H), 1.23 (m, 28H), 0.86 (t, 3H). ^13^C NMR (500 MHz, DMSO-d_6_, ppm): 172.98, 172.89, 156.12, 152.02, 152.53, 152.39, 149.22, 149.17, 148.91, 139.57, 139.24, 139.11, 119.28, 119.00, 118.94, 88.02, 85.97, 83.96, 83.17, 73.49, 71.17, 71.14, 71.00, 65.44, 65.18, 61.92, 50.01, 31.31, 29.06, 28.91, 28.85, 28.83, 28.72, 24.65, 22.12, 13.99. ^31^P NMR (500 MHz, DMSO-d_6_, ppm): 1.31, 1.21. HRMS (ESI-) m/z Calculated for C_28_H_47_N_5_O_8_P_1_ (MH-): 612.3167, found 612.3171.

### 3′-*O*-benzyloxycarbonyl-thymidine-5′-(*O*-(benzyl stearate)-2-yl, *O*-benzyl) phosphate (32)

To the stirring solution of **15** (2 g, 5.31 mmol) and **12** (5 g, 8 mmol) in dry DCM (5 mL), was added 0.5 M solution of 1H-Tetrazole (53 mL, 26.57 mmol) drop wise at 0°C and the reaction mixture was stirred at rt for 4 h. Then the reaction mixture was cooled down to -78°C and hydrogen peroxide 35% (W/V, 15 mL) was added. After stirring for 5 min at -78°C, cooling bath was removed and the reaction mixture was allowed to stir at rt for 30 min. The reaction mixture is diluted with DCM (200 mL) and washed with 1 M phosphoric acid (100 mL), dilute sodium bicarbonate (100 mL) and with brine (100 mL), dried over sodium sulfate, filtered, concentrated *in vacuo*. The obtained pale yellow oil was purified by column chromatography using EtOAc as eluent to give the desired compound **32** as oil (4.35 g, 89%). H^1^ NMR (CDCl_3_, 300 MHz, ppm): δ 8.85 (br s, 1H, -NH), 7.38 (m, 15H, 3xAr), 6.38 (m, 1H), 5.20 (m, 7H), 4.89 (m, 1H), 4.30 (m, 3H), 1.87 (m, 5H), 1.25 (m, 29H), 0.89 (t, 3H). ^13^C NMR (CDCl_3_, 300 MHz, ppm): δ 171.15, 169.83, 169.73, 163.87, 163.78, 154.36, 154.33, 150.55, 150.51, 150.49, 150.47, 135.40, 135.36, 135.14, 135.08, 135.01, 134.93, 134.79, 134.72, 128.97, 128.93, 128.85, 128.76, 128.68, 128.51, 128.45, 128.14, 128.11, 127.99, 127.95, 111.86, 111.84, 111.63, 84.44, 82.60, 70.22, 70.17, 69.83, 67.41, 60.42, 37.26, 31.97, 29.75, 29.71, 29.67, 29.56, 29.40, 29.09, 24.67, 24.58, 22.74, 21.07, 21.05, 19.12, 14.24, 14.16, 12.40. ^31^P NMR (500 MHz, CDCl_3_, ppm): δ -1.00, -1.24, -1.80, -1.83. HRMS (ESI+) m/z Calculated for C_50_H_67_N_2_O_12_P_1_ (MH+): 919.4504, found 919.4512.

### Thymidine-5′-(*O*-stearic acid-2-yl) phosphate (33)

To a stirring solutions of **32** (2 g, 2.17 mmol) in THF: MeOH-1:1 (30 mL) was added Palladium on Charcoal (10%), and kept for stirring under hydrogen for 6 h at room temperature. After completion of the reaction, the reaction mixture is passed through celite pad and the celite pad is washed with THF-MeOH mixture (200 mL). The organic solvents were removed under vacuum, and the obtained white residue was purified by silica gel chromatography using DCM:MeOH:H_2_O-17:7:1. The organic solvents were removed with a rotavapor and the aqueous solvent was removed with a lyophilizer to get the desired product **33** as white solid (0.81 g, 61%). ^1^H NMR (300 MHz, D_2_O, ppm): δ 7.82 (d, 1H, NH), 6.28 (d, 1H, H-5), 4.55 (br m, 2H), 4.14 (br m, 3H), 2.37 (m, 2H, 5′-H), 1.94 (m, 3H, CH_3_), 1.77 (br s, 2H, CH_2_), 1.42 (br, 2H), 1.18 (m, 26H, 13xCH_2_), 0.81 (t, 3H, CH_3_). ^13^C NMR (300 MHz, D_2_O, ppm): δ 173.02, 165.43, 150.90, 136.63, 110.88, 110.77, 85.53, 84.89, 74.38, 70.84, 70.60, 64.69, 39.23, 38.98, 33.09, 31.58, 29.53, 29.40, 29.27, 29.09, 24.74, 22.27, 13.53, 11.69, 11.61. ^31^P NMR (300 MHz, D_2_O, ppm): δ -0.80, -0.95. HRMS (ESI+) m/z Calculated for C_28_H_49_N_2_O_10_P_1_ (MH-): 603.3051, found 603.3054.

### 2′,3′-*O*-bisbenzyloxycarbonyl, adenosine-5′-((*O*-benzyl stearate)-2-yl, *O*-benzyl) phosphate (35)

To the stirring solution of **16** (2.5 g, 4.668 mmol) and 0.5 M solution of 1H-Tetrazole (46.7 mL, 23.34 mmol) in anhydrous dichloromethane, was added **12** (0.88 g, 7 mmol) in dry dichloromethane, drop wise at 0°C and the reaction mixture was stirred at rt for 4 h. Then the reaction mixture was cooled down to -78°C and hydrogen peroxide 35% (W/V) (15 mL) was added. After stirring for 5 min at -78°C, cooling bath was removed and the reaction mixture was allowed to stir at room temperature for 30 min. The reaction mixture is diluted with DCM (200 mL) and washed with 1 M phosphoric acid (100 mL), 5% aqueous sodium bicarbonate (100 mL) and with brine (100 mL). Organic layer was separated and dried over sodium sulfate, filtered and concentrated *in vacuo*. The obtained pale yellow oil was purified by silica gel column chromatography with eluent EtOAc to give **35** (4.55 g, 90%) as oil. ^1^H NMR (500 MHz, CDCl_3_, ppm): δ 8.28 (d, 1H, H-2), 8.07 (d, 1H, H-8), 7.34-7.29 (m, 20H, Ar), 6.21 (dd, 1H), 5.92 (m, 1H), 5.85 (br s, 2H), 5.65 (m, 1H), 5.18 (m, 1H), 5.12 (m, 6H), 5.87(m, 1H), 4.38 (m, 2H), 1.78 (m, 2H), 1.25 (m, 28H), 0.89 (t, 3H, CH_3_). ^13^C NMR (500 MHz, CDCl_3_, ppm): δ 170.13, 155.65, 154.09, 153.73, 153.47, 150.02, 139.38, 139.28, 135.27, 134.75, 134.59, 128.88, 128.85, 128.77, 128.71, 128.67, 128.600, 128.52, 128.11, 127.98, 127.92, 120.13, 85.63, 80.64, 76.17, 76.06, 73.98, 73.79, 70.63, 70.57, 69.83, 67.43, 66.2633.02, 32.97, 32.05, 29.83, 29.79, 29.66, 29.48, 29.45, 29.15, 24.64, 24.59, 22.81, 14.24. ^31^P NMR (500 MHz, CDCl_3_):-1.57,-1.61, -2.09. HRMS (ESI+) m/z Calculated for C_58_H_72_N_5_O_13_P_1_ (MH+): 1078.4936, found 1078.4946.

### Adenosine-5′-(*O*-stearic acid-2-yl) phosphate (36)

To a solution of **35** (2 g, 1.85 mmol) in THF, K_2_CO_3_ (0.52 g, 37 mmol) and water (2 mL) was added. To this, Pd (10%) on Charcoal is added and kept for stirring at room temperature under hydrogen for 72 h. After the completion of the reaction, the mixture is filtered on celite 545, filtrate was washed with THF:Water-1:1. The filtrate was concentrated *in vacuo* and purified by silica gel column chromatography (DCM:MeOH:H_2_O-17:7:1) to obtain the potassium salt of **33** as white solid (0.62 g, 53%). ^1^H NMR (500 MHz, CDCl_3_, ppm): δ 8.4 (br s, 1H, H-2), 8.13 (s, 1H, H-8), 7.21 (br s, 2H, -NH_2_), 5.92 (d, 1H, H-1′), 5.32 (m, 1H), 4.61 (br, 1H), 4.21 (br s, 1H), 4.04 (s, 1H), 3.90 (br s, 1H), 2.08 (s, 1H), 1.68 (br, 1H), 1.52 (br, 1H), 1.22 (m, 28H), 0.86 (t, 3H, CH_3_).

### 3′-O-benzyloxycarbonyl, deoxyadenosine-5′-(O-benzyl, O-(benzyl stearate)-2-yl) phosphate (38)

To the stirring solution of **14** (0.3 g, 0.77 mmol) and **13** (0.734 g, 1.16 mmol) in 5 mL dry DCM was added 0.5 M solution of 1H-tetrazole (12.5 mL, 6.22 mmol) drop wise at 0°C, and the reaction mixture was stirred at room temperature for 12 h. Then the reaction mixture was cooled down to -78°C and hydrogen peroxide 35% (W/V) was added. After stirring for 5 min at -78°C, cooling bath was removed and the reaction mixture was allowed to stir at rt for 30 min. The reaction mixture is diluted with DCM and washed with 1 M phosphoric acid, 5% aq. sodium bicarbonate and with brine, dried over sodium sulfate, filtered and concentrated on vacuo, and purified by column chromatography (EtOAc) which gives **38** as an oil. ^1^H NMR (300 MHz, CDCl_3_, ppm): δ7.37-7.28 (m, 15H), 6.45 (m, 2H), 5.31 (d, 1H), 5.13-4.98 (m, 6H, 3 × CH_2_), 4.29-4.20 (m, 1H), 2.78-2.69 (m, 1H), 2.68-2.54 (m, 1H), 1.65 (m, 2H, CH_2_), 1.25 (m, 26H, 13xCH_2_), 0.89 (t, 3H, CH_3_). ^13^C NMR (300 MHz, CDCl_3_, ppm): δ 169.89, 155.84, 154.25, 153.16, 149.69, 138.63, 135.73, 134.76, 128.77, 128.69, 128.57, 128.52, 128.40, 128.35, 128.26, 128.24, 128.13, 127.99, 127.96, 127.88, 127.81, 119.89, 84.08, 83.97, 82.93, 82.82, 78.32, 78.25, 76.52, 76.45, 76.38, 70.12, 69.62, 69.54, 69.46, 66.63, 40.15, 37.46, 35.25, 31.91, 29.69, 29.65, 29.54, 29.43, 29.41, 29.35, 29.28, 24.79, 22.68, 14.12. ^31^ P NMR (500 MHz, CDCl_3_, ppm): δ -1.92, -1.98, -2.17, -2.31. HRMS (ESI+) m/z Calculated for C_50_H_66_N_5_O_10_P_1_ (MH+): 928.4619, found 928.4615.

### 2′-deoxyadenosine-5′-(O-stearic acid-3-yl) phosphate (39)

To a stirring solution of **38** (0.2 g, 0.21 mmol) in THF, K_2_CO_3_ (60 mg, 4.31 mmol) and water (2 mL) is added. To this Pd (10%) on charcoal is added and kept for stirring at rt under hydrogen for 12 h. After completion, the reaction mixture is filtered on celite-545 and the solvent is evaporated and purified by column chromatography (DCM:MeOH:H_2_O-17:7:1) to obtain the potassium salt of **39** as white solid. ^1^H NMR (500 MHz, D_2_O, ppm): δ 8.48 (d, 1H), 8.09 (d, 1H), 6.42 (d,1H), 4.61 (br s, 1H), 4.26 (br s, 1H), 4.16 (br m, 2H, -NH_2_), 2.64 (m, 3H), 1.68 (m, 2H), 1.25 (m, 2H), 1.31-1.22 (m,2H), 1.01 (m, 18H, CH_2_), 0.66 (t, 3H, CH_3_). ^13^C NMR(500 MHz, D_2_O, ppm): 176.53, 154.04, 150.78, 147.64, 139.41, 117.66, 85.66, 83.89, 74.11, 70.77, 70.49, 64.73, 61.14, 58.56, 41.62, 40.05, 39.94, 35.24, 35.05, 33.61, 31.40, 29.34, 29.23, 28.91, 28.02, 24.66, 22.09, 13.37. ^31^P NMR(500 MHz, D_2_O, ppm): δ -1.04, -1.12. HRMS (ESI-) m/z Calculated for C_28_H_48_N_5_O_8_P_1_ (MH-): 612.3167, found: 612.3173.

### 3′-*O*-benzyloxycarbonyl thymidine 5′-(*O*-(benzyl stearate)-3-yl, *O*-benzyl)-phosphate (41)

To a stirring solution of **15** (0.3 g, 0.77 mmol) and **13** (0.734 g, 1.16 mmol) in 5 mL dry DCM was added 0.5 M solution of 1H-tetrazole (12.5 mL, 6.22 mmol) drop wise at 0°C, and the reaction mixture was stirred at room temperature for 12 h. Then the reaction mixture was cooled down to -78°C and hydrogen peroxide 35% (W/V) was added. After stirring for 5 min at -78°C, cooling bath was removed and the reaction mixture was allowed to stir at rt for 30 min. The reaction mixture is diluted with DCM and washed with 1 M phosphoric acid, 5% aq. sodium bicarbonate and with brine, dried over sodium sulfate, filtered and concentrated on vacuo, purified by column chromatography (EtOAc) which gives **41** as an oil. ^1^H NMR (500 MHz, CDCl_3_, ppm): δ 9.27 (br s, 1H, NH), 7.43-7.31 (m, 15H, 3 × Ar-H), 6.37 (m, 1H), 5.16 (s, 2H, Ar-CH_2_), 5.12-5.03 (m, 4H, Ar-CH_2_), 4.86 (m, 1H), 4.18 (m, 2H), 2.74-2.64 (m, 2H), 2.36 (m, 1H),2.03 (m, 1H), 1.89 (d, 2H), 1.69 (m, 2H), 1.25 (m, 26H, -CH_2_), 0.89 (t, 3H, CH_3_). ^1^H NMR (300 MHz, CDCl_3_, ppm): δ 169.87, 163.75, 154.36, 150.49, 135.63, 135.59, 135.11, 135.02, 134.76, 134.73, 128.85, 128.76, 128.68, 128.63, 128.50, 128.46, 128.36, 128.28, 128.09, 128.01, 127.95, 111.81, 111.73, 84.52, 84.44, 82.59, 82.48, 78.07, 78.02, 70.22, 70.20, 69.87, 69.80, 69.70, 69.63, 67.13, 67.06, 67.00, 66.72, 66.68, 40.25, 40.18, 40.12, 37.28, 37.20, 35.39, 31.97, 29.75, 29.71, 29.69, 29.60, 29.51, 29.41, 29.38, 24.91, 24.85, 22.71, 14.18, 12.44. ^31^P NMR(300 MHz, CDCl3): δ -1.68, -1.86, -2.06, -2.25. HRMS (ES+) m/z Calculated for C_50_H_67_N_2_O_12_P_1_ (MH+): 919.4504, found 919.4498.

### Thymidine-5′-(*O*-stearic acid-3-yl)-phosphate (42)

To the stirring solutions of **41** (2 g, 2.17 mmol) in THF:MeOH-1:1 (30 mL) was added Pd (10%) on charcoal, and kept for stirring under hydrogen for 6 h at room temperature. After completion of the reaction (monitored by TLC), the reaction mixture is passed through celite pad and the celite pad is washed with THF-MeOH mixture (200 mL). The organic solvents were removed under vacuum, and the obtained white residue was purified by silica gel chromatography using DCM:MeOH:H_2_O-17:7:1. The organic solvents were removed in the rotavapor and the aqueous solvent was removed by lyophilization to obtain the desired product **42** as white solid (0.94 g, 71%). ^1^H NMR (500 MHz, D_2_O, ppm): δ 7.84 (br m, 1H), 6.32 (br m, 1H), 4.60 (br m, 2H), 4.16 (m, 3H), 2.61 (m, 2H), 2.39 (m, 2H), 1.94 (s, 3H), 1.72 (m, 2H), 1.21 (m, 26H, CH_2_), 0.83 (s, 3H, CH_3_). ^13^C NMR (500 MHz, D_2_O, ppm): δ 174.93, 165.58, 151.16, 136.86, 111.04, 85.69, 85.20, 84.89, 73.60, 71.08, 70.80, 65.06, 64.67, 40.54, 39.33, 35.21, 31.78, 29.70, 29.60, 29.28, 24.80, 22.46, 13.74, 11.86. ^31^P NMR(500 MHz, CDCl3): δ -1.13, -1.15. HRMS (ES+) m/z Calculated for C_28_H_49_N_2_O_10_P_1_ (MH-): 603.3051, found 603.3048.

### 2′,3′-*O*-dibenzyloxycarbonyl, adenosine-5′-(*O*-(benzyl stearate)-3-yl, *O*-benzyl) phosphate (44)

To a stirring solution of **16** (2 g, 3.73 mmol) and **13** (2.81 g, 4.48 mmol) in dry DCM was added 0.5 M solution of 1H-tetrazole (0.5 M in dry acetonitrile (75 ml, 37.34 mmol) dropwise at 0°C and the reaction mixture was stirred at rt for 12 h. Then the reaction mixture was cooled down to -78°C and hydrogen peroxide 35% was added. After stirring for 5 min at -78°C, cooling bath was removed and the reaction mixture was allowed to stir at rt for 30 min. The reaction mixture is diluted with DCM and washed with 1 M phosphoric acid, dilute sodium bicarbonate and with brine, dried over sodium sulfate, filtered and concentrated *in vacuo*, purified by column chromatography with eluent EtOAC to give **44** as oil. ^1^H NMR (500 MHz, CDCl_3_, ppm) : δ 8.27 (s, 1H, H-2), 8.01-7.97 (s, 1H, H-8), 7.33-7.25 (m, 20H, 4xAr-H), 6.19 (d, 1H), 5.93-5.91 (m, 3H), 5.62 (m, 1H), 5.12-5.01 (m, 8H, CH_2_-Ar), 4.81 (m, 1H), 4.38 (m, 1H), 4.30-4.28 (m, 1H), 4.24-4.22 (m, 1H), 2.7-2.70 (m, 1H), 2.61-2.57 (m, 1H), 2.21 (br s, 1H), 1.67-1.60 (m, 2H), 1.25 (m, 26H), 0.89 (t, 3H, CH_3_).^13^C NMR (500 MHz, CDCl_3_, ppm): 170.05, 155.70, 154.00, 153.72, 153.48, 149.90, 139.26, 135.78, 134.69, 134.54, 128.85, 128.75, 128.70, 128.64, 128.54, 128.52, 128.40, 128.06, 127.98, 120.16, 85.83, 85.79, 85.63, 80.65, 80.59, 76.66, 73.79, 70.65, 70.53, 69.67, 66.64, 65.94, 40.22, 35.31, 32.03, 29.81, 29.77, 29.68, 29.61, 29.54, 29.47, 29.40, 24.86, 22.79, 14.23. ^31^P NMR (500 MHz, CDCl_3_, ppm): δ (q -1.96, -2.07, -2.25, -2.39). HRMS(ES+) m/z Calculated for C_58_H_72_N_5_O_13_P_1_ (MH+): 1078.4937, found 1078.4946.

### Adenosine-5′-(*O*-stearic acid-3-yl)-phosphate (45)

To the solution of **44** (0.3 g, 0.27 mmol) in THF, K_2_CO_3_ (77 mg, 0.55 mmol) and water 2 mL was added. To this Pd (10%) on charcoal is added and held for stirring at rt under hydrogen for 12 h. After completion of the reaction, the reaction mixture is filtered on celite and the solvent is evaporated and purified by column chromatography (DCM: MeOH: H_2_O-17:7:1) to obtain the potassium salt of **45** as white solid. ^1^H NMR (500 MHz, D_2_O, ppm): δ 8.42 (d, 1H, H-2), 8.12 (s, 1H, H-8), 7.24 (br s, 2H, -NH_2_), 5.91 (d, 1H), 4.53 (m, 1H), 4.19 (m, 2H), 4.01 (br s, 1H), 3.93 (m, 2H), 2.68 (m, 1H), 2.40 (ddd, 1H), 1.91 (m, 1H), 1.60 (m, 1H), 1.45 (m, 1H), 1.23 (m, 26H, CH_2_), 0.86 (t, 3H, CH_3_). ^13^C NMR (500 MHz, D_2_O, ppm): δ 172.50, 165.45, 155.98, 152.55, 149.58, 139.32, 139.19, 127.96, 126.94, 118.83, 86.93, 83.74, 74.01, 73.88, 71.17, 70.82, 70.64, 67.48, 64.73, 64.41, 60.48, 53.30, 47.85, 43.37, 42.56, 35.52, 31.28, 29.04, 28.69, 24.89, 22.08, 13.95. ^31^P NMR (500 MHz, D_2_O): δ -0.69. HRMS(ES-) m/z Calculated for C_28_H_48_N_5_O_9_P_1_ (MH-): 628.3116, found 594.3061.

### 9-((*O*-benzyl, *O*-(*O*-benzyl stearate)-2-yl-5′ phospho), 3′-*O*-benzyl, 2′-*O*-benzyloxycarbonyl xylofuranosyl) adenine (46)

To a stirred solution of **27** (1.2 g, 24.41 mmol) and **12** (2.3 g, 36.62 mmol) in 12 mL dry DCM was added 0.5 M solution of 1H-Tetrazole (24.41 mL, 122.07 mmol) drop wise at 0°C, and the reaction mixture was stirred at room temperature for 12 h. Then the reaction mixture was cooled down to -78°C and hydrogen peroxide 35% (W/V) was added. After stirring for 5 min at -78°C, cooling bath was removed and the reaction mixture was allowed to stir at room temperature for further 30 min. The reaction mixture is diluted with DCM (150 mL) and washed with 1 M phosphoric acid (100 mL), 5% aq. sodium bicarbonate (100 mL) and with brine (80 mL), dried over sodium sulfate, filtered and concentrated *in vacuo*. The obtained residue was purified by column chromatography (EtOAc) giving **46** (100 mg, 4%) as an oil. ^1^H NMR (300 MHz, CDCl_3_, ppm): δ 8.34 (s, 1H, H-2), 8.09 (m, 1H, H-8), 7.38-7.21 (m, 20H, Ar-H), 6.33 (m, 1H), 5.93 (br s, 1H), 5.41 (br s, 1H), 5.20-5.02 (m, 6H, CH_2_-Ar), 4.83 (m, 1H), 4.67 (m, 1H), 4.54 (m, 1H), 4.44 (m, 3H), 4.09 (br m, 1H), 1.80 (m, 2H), 1.25 (m, 26H, CH_2_), 0.87 (m, 3H, CH_3_). ^13^C NMR (300 MHz, CDCl_3_, ppm): δ 170.03, 155.58, 153.84, 153.37, 149.69, 139.24, 139.15, 136.38, 136.27, 135.73, 135.65, 135.27, 134.46, 129.04, 128.85, 128.67, 128.57, 128.47, 128.42, 128.19, 128.12, 128.06, 127.92, 119.54, 87.38, 87.33, 82.60, 82.52, 81.34, 81.23, 81.16, 80.02, 79.94, 76.04, 75.97, 72.40, 70.80, 69.87, 69.82, 69.72, 69.65, 67.25, 65.37, 65.30, 65.21, 65.10, 65.03, 33.06, 32.97, 32.02, 29.80, 29.75, 29.71, 29.60, 29.45, 29.41, 29.11, 24.60, 22.78, 14.22. ^31^P NMR (300 MHz, CDCl_3_, ppm): δ -1.58, -1.68, -1.82, -1.91. HRMS (ESI+) m/z Calculated for C_57_H_72_N_5_O_11_P_1_ (MH+):1034.5038, found: 1034.5048.

### 9-xylofuranosyl adenine-5′-(*O*-stearic acid-2-yl) phosphate (47)

To a solution of **46** (100 mg, 0.09 mmol) in THF, K_2_CO_3_ (27 mg, 0.18 mmol) and water (2 mL) is added. To this Palladium (10%) on charcoal (10 mg) is added and held for stirring at rt under hydrogen for 12 h. After the completion of the reaction, the mixture is filtered on celite and the solvent is removed *in vacuo*. The obtained residue was purified by column chromatography with the eluent (DCM:MeOH:H_2_O-17:7:1) to obtain the potassium salt of **47** as white solid. ^1^H NMR (300 MHz, CDCl_3_, ppm): δ 8.35 (m, 1H, H-2), 8.13 (s, 1H, H-8), 7.31 (d, 2H), 5.91 (br s, 1H), 4.31 (m, 2H), 4.12 (m, 2H), 4.05 (m, 1H), 3.87 (m, 1H), 3.80-3.61 (m, 1H), 1.63 (m, 2H), 1.19 (m, 24H, CH_2_), 0.85 (t, CH_3_). ^13^C NMR (300 MHz, CDCl_3_, ppm): δ 156.06, 155.96, 152.43, 149.02, 148.78, 139.79, 118.44, 89.42, 83.62, 82.01, 80.93, 75.05, 74.87, 60.64, 59.27, 54.96, 31.34, 29.20, 28.78, 28.28, 22.12, 13.92. ^31^P NMR (300 MHz, CDCl_3_, ppm): δ 1.67, 1.36. HRMS (ESI-) m/z Calculated for C_28_H_48_N_5_O_9_P_1_ (MH-): 628.3116, found: 628.3123.

### 9-((*O*-benzyl, *O*-(*O*-benzyl stearate)-3-yl-5′ phospho), 3′-*O*-benzyl, 2′-*O*-benzyloxycarbonyl xylofuranosyl) adenine (48)

To a stirred solution of **27** (1.2 g, 2.44 mmol) and **13** (2.3 g, 3.66 mmol) in 20 mL dry DCM was added 0.5 M solution of 1H-Tetrazole (54 mL, 24.4 mmol) drop wise at 0°C, and the reaction mixture was stirred at room temperature for 12 h. Then the reaction mixture was cooled down to -78°C and hydrogen peroxide 35% (W/V) was added. After stirring for 5 min at -78°C, cooling bath was removed and the reaction mixture was allowed to stir at rt for 30 min. The reaction mixture is diluted with DCM and washed with 1 M phosphoric acid, 5% aq. sodium bicarbonate and with brine, dried over sodium sulfate, filtered and concentrated *in vacuo*, purified by column chromatography (EtOAc) which gives **48** as an oil. ^1^H NMR (500 MHz, CDCl_3_, ppm): δ 8.33 (s, 1H, H-2), 8.10 (m, 1H, H-8), 7.38-7.19 (m, 20H, 4 × Ar), 6.33 (d, 1H, H-1′), 6.14 (br s, 2H, -NH_2_), 5.42 (br s, 1H), 5.19 (m, 2H), 5.08-5.00 (m, 4H), 4.83 (m, 1H), 4.66 (dd, 1H), 4.54 (dd, 1H), 4.38 (m, 1H), 4.35 (m, 2H), 4.10 (m, 1H), 3.55-3.46 (m, 1H), 2.77-2.72 (m, 1H, H-5′), 2.63-58 (m, 1H, H-5″), 1.66 (m, 2H, CH_2_), 1.25 (m, 28H, CH_2_), 0.89 (t, 3H, CH_3_). ^13^C NMR (500 MHz, CDCl_3_, ppm): δ 169.91, 155.68, 153.75, 153.22, 149.53, 139.02, 136.24, 135.82, 135.62, 134.36, 128.95, 128.75, 128.63, 128.58, 128.57, 128.54, 128.36, 128.27, 128.12, 128.10, 128.00, 127.92, 128.86, 119.41, 87.25, 82.44, 81.24, 79.91, 76.28, 72.27, 70.69, 69.47, 66.54, 64.92, 40.33, 35.26, 31.94, 29.71, 29.71, 29.68, 29.64, 29.56, 29.45, 29.37, 29.31, 24.76, 22.98, 22.71, 14.16. HRMS (ES-) m/z Calculated for C_57_H_72_N_5_O_11_P_1_ (MH+): 1034.5038, found: 1034.5026.

### β-D-xylofuranosyladenine-5′-(*O*-stearic acid-3-yl)-phosphate (49)

To the solution of **48** (0.2 g, 0.21 mmol) in THF, K_2_CO_3_ (60 mg, 4.31 mmol) and water (2 mL) is added. To this Pd (10%) on charcoal is added and held for stirring at rt under hydrogen for 12 h. After the completion of the reaction, the mixture is filtered on celite-545 and the solvent is evaporated and the residue is purified on column chromatography (DCM:MeOH:H_2_O-17:7:1) to obtain the potassium salt of **49** as white solid. H^1^ NMR (500 MHz, DMSO-d6, ppm): δ 8.26 (m, 1H), 8.11 (d, 1H, H-8), 5.98 (br s, 1H), 4.63 (m, 2H), 4.46 (m, 2H), 4.25 (m, 2H), 3.60 (t, 1H), 2.44 (m, 2H), 1.78 (t, 1H), 1.55 (m, 2H), 1.06 (m, 26H, CH_2_), 0.74 (t, 3H, CH_3_). ^13^C NMR (600 MHz, DMSO-d_6_, ppm): δ 179.31, 162.09, 154.98, 152.10, 152.03, 148.07, 147.75, 139.95, 139.86, 118.24, 118.09, 89.68, 88.46, 81.43, 81.39, 80.54, 80.11, 79.59, 75.66, 75.53, 74.57, 62.98, 62.75, 61.24, 48.57, 44.01, 35.17, 33.73, 31.45, 29.33, 29.24, 28.93, 28.12, 24.69, 24.63, 22.97, 22.17, 13.51. ^31^P NMR (500 MHz, D_2_O, ppm): -0.94, -1.09. HRMS (ES-) m/z Calculated for C_28_H_48_N_5_O_9_P (MH+): 628.3117, found: 628.3119.

### 2′-deoxyadenosine-5′-(*N*-(methyl stearate)-2-yl)-phosphoramidate (51)

In a 100 mL two neck flask, 2′-deoxyadenosine 5′-O-monophosphate (0.3 g, 0.9 mmol) and methyl α-aminostearate **50** (1.42 g, 4.52 mmol) were dissolved in a mixture of *t-*butanol and water (5:1). Triethylamine (0.5 mL), and a freshly prepared solution of N, N’-dicyclohexylcarbodiimide (DCC) in t-butanol (0.5 g/mL) was added to the reaction mixture under argon atmosphere, and the reaction mixture was allowed to reflux for 2 h. The progress of the reaction was monitored by TLC. Another 5 eq of DCC was added to the reaction mixture, and was continued refluxed under argon for 1 h. Upon completion, the reaction mixture was cooled down to room temperature and the solvent was concentrated under vacuo. The residue obtained was purified by silica gel column chromatography (DCM:MeOH:H_2_O-17:7:1) to obtain **51** (0.44 g, 77%) as white solid. ^1^H NMR (500 MHz, DMSO, ppm): δ 8.38 (d, 1H, H-2), 8.12 (s, 1H, H-8), 7.22 (br s, 2H), 6.35 (t, 1H), 4.41 (s, 1H), 3.92 (br s, 1H), 3.78-3.74 (m, 2H), 3.67 (m, 1H), 3.55 (s, 2H), 3.52 (s, 1H), 3.02 (dd, 3H), 2.69-2.63 (m, 1H), 1.47 (m, 2H), 1.22-1.14 (m, 28H, 14xCH_2_), 0.85 (t, 3H, CH_3_). ^13^C NMR (500 MHz, DMSO, ppm): δ 175.11, 155.95, 152.50, 149.18, 139.20, 118.86, 117.45, 82.97, 71.34, 64.14, 54.47, 51.28, 45.20, 31.26, 29.01, 28.87, 28.73, 28.67, 24.94, 22.07, 13.93, 8.46. ^31^P NMR (500 MHz, DMSO): δ 4.51. HRMS (ESI+) m/z Calculated for C_29_H_50_N_6_O_7_P_1_(MH-): 625.3483 found 625.3453.

### 2′-deoxyadenosine-5′-(*N*-stearic acid-2-yl)-phosphoramidate (52)

A solution of **51** (0.4 g, 0.63 mmol) in MeOH/H_2_O (4:1 v/v, 3 mL, containing 0.4 M of NaOH (0.051 g, 1.27 mmol) was stirred at room temperature under nitrogen for 2 h. After completion of the reaction, the solvents were removed under reduced pressure. The resulted crude reaction mixture was purified by silica gel chromatography (DCM:MeOH:H_2_O-17:7:1) to obtain **52** (0.28 g, 71%) as white solid. ^1^H NMR (500 MHz, DMSO, ppm): δ 8.42 (d, 1H, H-2), 8.11 (d, 1H, H-8), 7.23 (br s, 2H, -NH_2_), 6.37 (m, 1H), 4.44 (d, 1H), 3.97 (m, 1H), 3.82-3.71 (m, 3H), 2.69 (m, 1H), 2.27 (m, 1H), 1.65 (br s, 1H), 1.21 (m, 28H), 0.85 (t, 3H). ^13^C NMR (500 MHz, DMSO, ppm): δ 176.18, 156.08, 152.59, 149.31, 139.31, 119.01, 86.45, 83.21, 71.62, 64.31, 64.09, 55.13, 54.78, 31.87, 31.39, 29.14, 25.90, 25.87, 22.20, 14.06. ^31^P NMR (500 MHz, DMSO): δ 7.08, 6.96. HRMS (ES-) m/z Calculated for C_28_H_49_N_6_O_7_P_1_ (MH-): 611.3327, found: 611.3323.

### Thymidine-5′-(*N*-(methyl stearate)-2-yl)-phosphoramidate (53)

In a 100 mL two neck flask, 2′-deoxythymidine 5′-O-monophosphate (0.5 g, 1.55 mmol) and methyl α-aminostearate **50** (2 g, 6.20 mmol) were dissolved in a mixture of *t-*butanol and water (5:1). Triethylamine (0.5 mL), and a freshly prepared solution of N, N’-dicyclohexylcarbodiimide (DCC, 1.6 g, 7.75 mmol) in *t-*butanol (0.5 g/mL) was added to the reaction mixture under argon atmosphere, and the reaction was allowed to reflux for 2 h. Another 5 eq of DCC was added to the reaction mixture which was refluxed for 1 h. Upon completion, the reaction mixture was cooled down to room temperature and the reaction mixture was concentrated *in vacuo*. The residue obtained was purified by silica gel column chromatography (DCM:MeOH:H_2_O-17:7:1) to obtain **53** (0.69 g, 72%) as white solid. ^1^H NMR (500 MHz, DMSO): δ 10.82 (br s, 1H, -NH), 7.28 (d, 1H), 5.70 (m, 1H), 5.24 (s, 1H), 3.76 (br s, 1H), 2.67 (s, 2H), 1.56 (m, 2H), 1.32 (br s, 2H), 0.71 (m, 28H, 14xCH_2_), 0.34 (m, 3H, CH_3_).

^13^C NMR (300 MHz, DMSO, ppm): δ 175.26, 163.79, 150.50, 136.26, 109.62, 86.03, 85.92, 83.74, 70.97, 63.82, 54.54, 54.44, 51.19, 49.19, 45.10, 34.30, 31.28, 30.21, 29.03, 28.75, 28.70, 25.01, 24.62, 23.83, 22.07, 13.87, 12.06, 8.36. ^31^P NMR (300 MHz, DMSO, ppm): δ 3.70. HRMS (ES-) m/z Calculated for C_29_H_52_N_3_O_9_P_1_ (MH-): 616.3368, found: 616.3371.

### Thymidine-5′-(*N*-stearic acid-2-yl)-phosphoramidate (54)

A solution of **53** (0.5 g, 0.8 mmol) in MeOH/H_2_O (4:1-v/v), was added 0.4 M NaOH (0.065 g, 1.61 mmol), and the mixture was stirred at room temperature under nitrogen for 2 h. The solvent was removed under reduced pressure. The resulting crude material was purified by chromatography (DCM:MeOH:H_2_O-17:7:1) to obtain **54** (0.34 g, 69%) as white solid. ^1^H NMR (500 MHz, DMSO): δ11.24 (s, 1H), 7.84 (s, 1H), 6.22 (q, 1H), 5.36 (br s, 1H), 4.31 (d, 1H), 3.86-3.72 (m, 1H), 2.12-2.01 (m, 2H), 1.80 (m, 1H), 1.76 (), 1.23 (m, 28H), 0.86 (t, 3H, CH_3_). ^13^C NMR (500 MHz, DMSO): 176.13, 163.89, 150.58, 136.39, 109.79, 86.12, 83.84, 71.35, 71.00, 31.38, 29.12, 26.03, 25.85, 22.18, 14.05, 12.16, 12.12. ^31^P NMR (500 MHz, DMSO): δ 7.29, 7.00. HRMS (ES-) m/z Calculated for C_28_H_49_N_3_O_9_P_1_ (MH-): 602.3211, found 602.3210.

### Bodipy (55)

8-S-Methyl Bodipy (120 mg, 0.5 mmol, prepared according to ref. [41]) is dissolved in DMSO/DCM (1/1; v/v, 5 ml) and mixed with taurine (60 mg, 0.5 mmol) and NaHCO_3_ (42 mg, 0.5 mmol). The resulting mixture is stirred at room temperature until TLC indicates complete consumption of the starting material, and the formation of a highly polar compound displaying blue fluorescence. The reaction mixture is diluted with water (10 ml) and dichloromethane (10 ml) and extracted. The aqueous layer is collected and lyophilized to yield the desired product **55** as a pale yellow solid. ^1^H NMR(300 MHz, DMSO, ppm): δ 7.47 (br s, 1H, -CH), 7.36 (d, 1H, -CH), 7.24 (br s, 1H, -CH), 7.11 (d, 1H, -CH), 6.49-6.47 (m, 1H, -CH), 6.31-6.29 (m, 1H, -CH), 3.99 (t, 2H, -CH_2_), 2.92 (t, 2H, -CH_2_). ^13^C NMR (500 MHz, DMSO, ppm): δ 147.79, 132.15, 129.05, 122.45, 122.18, 114.57, 113.77, 112.42, 40.42.

### Vesicles preparation and visualization

Nucleolipid aggregate/vesicle formation was performed by dissolving the nucleolipids in water or DMSO and dilute with water or THF/dioxane (1:1) in a glass vial. To this solution Bodipy fluorescent dye either in water, THF or chloroform was added, vortexed for 10 seconds to stimulate vesicle formation and set aside for 5 min. The pH of the nucleolipids emulsion is found to be 6.82. If the pH was lowered further, the emulsion appeared opalescent. An aliquot of the mixture (100 μL) was pipetted out from this reaction mixture and spin coated at 3000 rpm on a microscope glass plate for 2 min, resulting in a thin layer adequate for optical microscopy.

Vesicles formed in presence of Bodipy fluorescent dye were monitored under fluorescent microscopy and the images were recorded with Olympus Fluoview FV1000 by carrying out excitation wavelength readings at 532 nm with 100 × zoom. Two fluorescent dyes were used for the vesicle encapsulation: aqueous soluble dye **55** (soluble in both water) and organic soluble dye **56** (chloroform/THF soluble), which were used at a concentration of 0.5 μM.

### Stability study by NMR

Samples were prepared in D_2_O or DMSO and the pD of the sample was adjusted by the addition of a small volume (a few μL) of HCl or NaOH solutions in D_2_O (0.1 M). ^31^P NMR was used to study the stability of the nucleolipids in acidic (pH4) and in base (pH12) environment. One-dimensional ^31^P spectra were used as a fast screening experiment to monitor degradation of the conjugates. Two-dimensional ^1^H-^31^P correlation spectra were used to characterize the ^31^P containing products formed by degradation of the nucleolipids. Correlations were established using a Proton-detected hetero-TOCSY experiment [24] with a DIPSI spinlock of 50 ms, allowing correlations of ^31^P resonances with several ^1^H resonances of adjacent spin systems.

## Conclusions

We have synthesized a series of amphiphiles in which the polar group consists of an adenine or thymine nucleotide and the lipid moiety is based on stearic acid. The nucleolipids are constructed from a phosphodiester or phosphoramidate bond formed between α- or β-hydroxyl group or the α-amino group present on the lipid moiety and the 5′-phosphate group of the nucleotide. These molecules have been analyzed for their potential to form vesicles in water. This can be considered as a model of a protocell with a shell containing covalently bond nucleotides, which may be used to establish an information system in the vesicle by polymerization. The functionalized lipid may function as leaving group for the polymerization reaction and the α (or β) carboxylic acid may catalyze phosphodiester cleavage.

The nucleolipids with a deoxyribose sugar moiety may form small or large vesicles, rod-like structures or vesicle aggregates. Some of these aggregates can be considered as intermediate forms in vesicle formation or division. It seems that a diversity of communication systems, by diffusion (in/out the vesicle) or exchange of material between vesicles (via vesicle fusion), between such vesicles exist. Suggesting that a protocell could stay out of equilibrium by diverse forms of material exchange.

However, we could not observe nucleotide polymerization or cyclic nucleotide formation of these nucleolipids, regardless of the sugar moiety that is investigated (deoxyribose, ribose, xylose). To unravel this observation, the chemical stability of the constructs was studied. While the nucleolipids containing β-hydroxy fatty acids are stable as well in base as in acid circumstances, others degraded in acidic conditions. Phosphoramidate nucleolipids hydrolyzed by P-N as well as P-O bond cleavage where the ratio between both pathways depends on the nucleobase. Diester constructs with a α-hydroxy stearic acid degraded exclusively by hydrolysis of the phosphorus to 5′-O-nucleoside ester. To summarize, among all investigated systems, only α-amino compounds have shown the desired bond breakage, the only problem being that the nucleophile is water and not the 2’- or 3’- hydroxyl groups. Favoring the intramolecular mechanism in order to promote polymerization, acyclic sugar moieties such as in GNA could be considered. Also their prebiotic relevance makes them ideal candidates to explore protocells capable of simultaneous core and shell replication [48]. As the compounds are too stable and harsh conditions would destruct the material itself, more reactive species (such as lipid imidazolates of nucleotides) need to be synthesized to further analyze the potential polymerization process. This research could be based on the original work of L. Orgel [49], in which he used phosphorimidazolate for nucleotide polymerization. Furthermore, quantitative investigation is in order to address the interesting (hydro)gelating properties of these new phosphodiester nucleolipids in acidic aqueous environment.

## Methods

All reactions were performed in an inert atmosphere (argon). Chemicals and solvents were purchased from Sigma-Aldrich, TCI Europe, and Alfa Aesar, and were used without further purification. Unless otherwise mentioned, each reaction vessel was oven dried prior to use. Column chromatography was performed using silica gel (63-200 mesh) obtained from the Sigma-Aldrich Company. Analytical thin-layer chromatography (TLC) was performed on Merck pre-coated aluminum plates (silica gel 60, F_254_) and visualized under 254-nm UV light. ^1^H, ^31^P and ^13^C NMR spectra were obtained on a Bruker 300 MHz and Bruker 500 MHz instrument at ambient temperature in CDCl_3_-d3, MeOD-d4, D_2_O and DMSO-d6. Chemical shifts are reported as values in parts per million (ppm) downfield from internal standard tetramethylsilane (δ = 0.0 ppm) from the residual solvent signal. Splitting patterns are designated as follows: s, singlet; d, doublet; t, triplet; m, multiplet; dd, doublet of doublet; br, broad peak. NMR signal assignment of sugar protons and carbons are numbered with a prime. ^31^P NMR chemical shifts are referenced to an external 85% H_3_PO_4_ standard (*d* =0.000 ppm). Mass spectra were performed on a Hewlett Packard MALDI-TOF spectrometer. Fluorescent microscopy images were recorded by Olympus Fluoview FV 1000 spectrometry.
